# MicroRNA-132 promotes fibroblast migration via regulating RAS p21 protein activator 1 in skin wound healing

**DOI:** 10.1038/s41598-017-07513-0

**Published:** 2017-08-10

**Authors:** Xi Li, Dongqing Li, Jakob D. Wikstrom, Andor Pivarcsi, Enikö Sonkoly, Mona Ståhle, Ning Xu Landén

**Affiliations:** 1 Unit of Dermatology and Venereology, Department of Medicine, Karolinska Institutet, Stockholm, Sweden; 2 Unit of Dermatology and Venereology, Karolinska University Hospital, Stockholm, Sweden

## Abstract

MicroRNA (miR)-132 has been identified as a top up-regulated miRNA during skin wound healing and its inhibition impairs wound repair. In a human *in vivo* surgical wound model, we showed that miR-132 was induced in epidermal as well as in dermal wound–edge compartments during healing. Moreover, in a panel of cells isolated from human skin wounds, miR-132 was found highly expressed in human dermal fibroblasts (HDFs). In HDFs, miR-132 expression was upregulated by TGF-β1. By overexpression or inhibition of miR-132, we showed that miR-132 promoted HDF migration. Mechanistically, global transcriptome analysis revealed that RAS signaling pathway was regulated by miR-132 in HDFs. We found that RAS p21 protein activator 1 (RASA1), a known target of miR-132, was downregulated in HDFs upon miR-132 overexpression. Silencing of RASA1 phenocopied the pro-migratory effect of miR-132. Collectively, our study reveals an important role for miR-132 in HDFs during wound healing and indicates a therapeutic potential of miR-132 in hard-to-heal skin wounds.

## Introduction

Wound healing is a fundamental physiological process to protect the integrity of the skin barrier. It is usually characterized as four sequential but overlapping phases, i.e. hemostasis (0- several hours after injury), inflammation (~1–3 days), proliferation (~4–21 days) and remodelling phases (~21 days–1 year)^1^. Impaired healing may result in chronic wounds, a major and rising health and economic problem worldwide. It is estimated that 1–2% of individuals in developed countries will experience a hard-to-heal wound during their life time^2^, already consuming 2–4% of the health care budget in these countries^3–5^. Despite extensive research, precise mechanisms underlying the pathology of chronic wounds are not fully understood, which impedes the development of effective treatment.

Dermal fibroblasts play pivotal roles during skin wound healing by forming granulation tissue, regulating angiogenesis, assisting re-epithelialization, producing and remodelling of extracellular matrix needed to restore dermis^6–8^. However, primary fibroblasts derived from the non-healing edge of chronic wounds exhibit diminished replicative^9–11^ and migratory capacities^12–14^. They also produce less growth factors e.g. FGF and VEGF^15^ and show reduced mitogenic response to PDGF and IGF stimulation^16^. In addition, abnormal excess production of proteinases by these fibroblasts results in a hyperproteolytic environment in chronic wounds^15, 17, 18^.

MicroRNAs (miRNAs) are ~22nt non-protein-coding RNAs (8). By binding to the 3′ untranslated region (UTR) of target mRNAs and incorporating with the RNA-induced silencing complex (RISC), miRNAs comply translational repression or degradation of its targets^19^. MiRNAs are widely acknowledged as important regulators in multiple biological processes and diseases. We have recently identified microRNA (miR)-132 as a top up-regulated miRNA during human skin wound healing^20^. Inhibition of miR-132 delays wound healing in mouse *in vivo* and human *ex vivo* wound models^20^. In epidermal keratinocytes, we found that miR-132 inhibits inflammation but promotes cell proliferation^20^. Considering the central role of fibroblasts in wound healing, and to get a more complete understanding of the role of miR-132 in wound repair, herein we clarified the regulation and function of miR-132 in dermal fibroblasts.

## Results

### MiR-132 expression is induced by TGF-β1 in dermal fibroblasts

**Figure 1:**
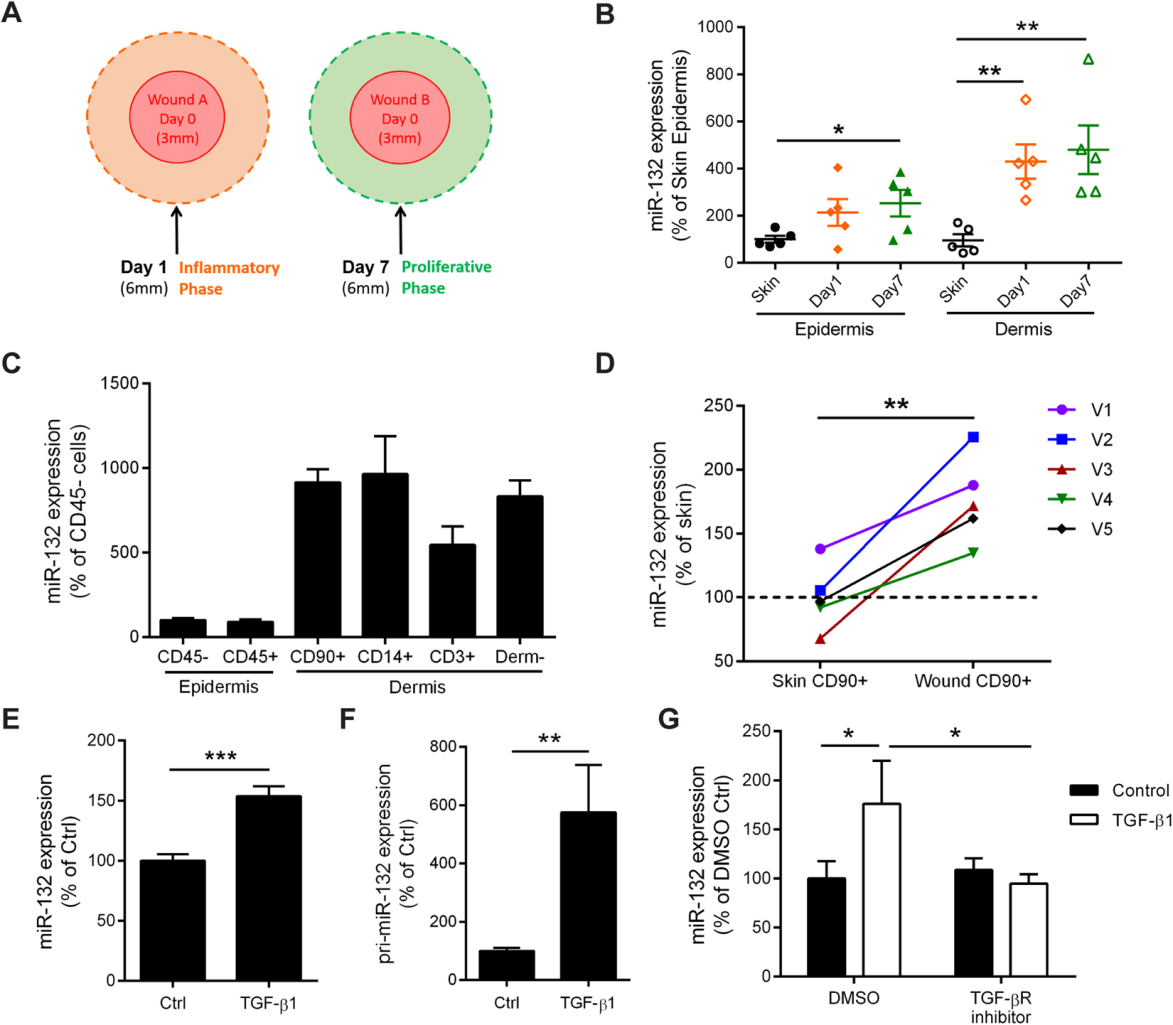
Expression and regulation of miR-132 in wounds. (**A**) The intact skin (Day 0), Day 1 and Day 7 wounds were collected from healthy donors. (**B**) MiR-132 expression was analyzed in the epidermis and dermis of the biopsies from donor 1–5, which were separated by laser capture microdissection. (**C**) QRT-PCR analysis of miR-132 expression in the cells isolated from the Day6 wounds of 5 donors, including CD45- and CD45 + cells from epidermis, and CD90 + , CD14 + , CD3 + and CD90-CD14-CD3- (Derm-) cells from dermis. (**D**) QRT-PCR analysis of miR-132 expression in dermal CD90 + cells from intact skin and Day6 wounds. V1–5 indicate 5 healthy volunteers. QRT-PCR analysis of miR-132 (**E**) or its primary precursor pri-miR-132 (**F**) in HDFs treated with 10 nM TGF-β1 for 48 hours (n = 3). (**G**) The TGF-β receptor inhibitor SB431542 was applied 15 minutes before adding TGF-β1 to HDFs, and miR-132 expression was analyzed 48 hours later (n = 3). The data are presented as mean ± s.e.m. in (**B**, **C**), as mean ± s.d. in (**E-G**). *P < 0.05, **P < 0.01 and ***P < 0.001 by Student’s t test.

To reveal the expression pattern of miR-132 during human skin wound healing, we made surgical wounds in 10 healthy donors and collected intact skin and wound-edge tissues at the inflammatory phase (one day after wounding) and at the proliferative phase (6 or 7 days after wounding) (Fig. 1A, Supplementary Table S1). Epidermal and dermal compartments of the biopsies from donor 1–5 were separated using laser capture microdissection. We found that during wound healing miR-132 was not only up-regulated in the epidermis as shown previously^20^, but also significantly increased in the dermis (Fig. 1B). Next, we isolated epidermal CD45- cells (mainly composed of keratinocytes) and CD45 + cells (leukocytes), dermal CD90 + cells (fibroblasts), CD14 + cells (macrophages), CD3 + cells (T cells) and CD90- CD14- CD3- cells from the Day 6 wounds of donor 6–10 using immunoselection. We found that miR-132 was highly expressed in human dermal fibroblasts (HDFs) in wounds (Fig. 1C). Interestingly, compared to the HDFs from the intact skin, miR-132 expression was significantly upregulated (1.76-fold change, P = 0.0074) in the wound HDFs (Fig. 1D).

TGF-β1 is a central cytokine in the wound healing cascade^21^. We have previously shown that TGF-β1 is up-regulated during normal skin wound healing and that it induces miR-132 expression in epidermal keratinocytes^20^. Here we found that TGF-β1 also significantly increased (1.53-fold change, P = 0.0072) the expression of miR-132 in HDFs (Fig. 1E). Moreover, we showed that TGF-β1 induced (5.75-fold change, P = 0.0071) the expression of miR-132 primary precursor (pri-miR-132), suggesting that TGF-β1 regulates transcription of the miR-132 gene (Fig. 1F). Inhibition of the TGF-β type I activin receptor-like kinase (ALK) receptor by a chemical inhibitor, SB431542, completely abolished TGF-β1-mediated induction of miR-132 (Fig. 1G). Together, our data suggest that the expression of miR-132 is upregulated in HDFs during wound healing, which may be due to the increased TGF-β signalling.

### Transcriptome analysis of dermal fibroblasts overexpressing miR-132

**Figure 2:**
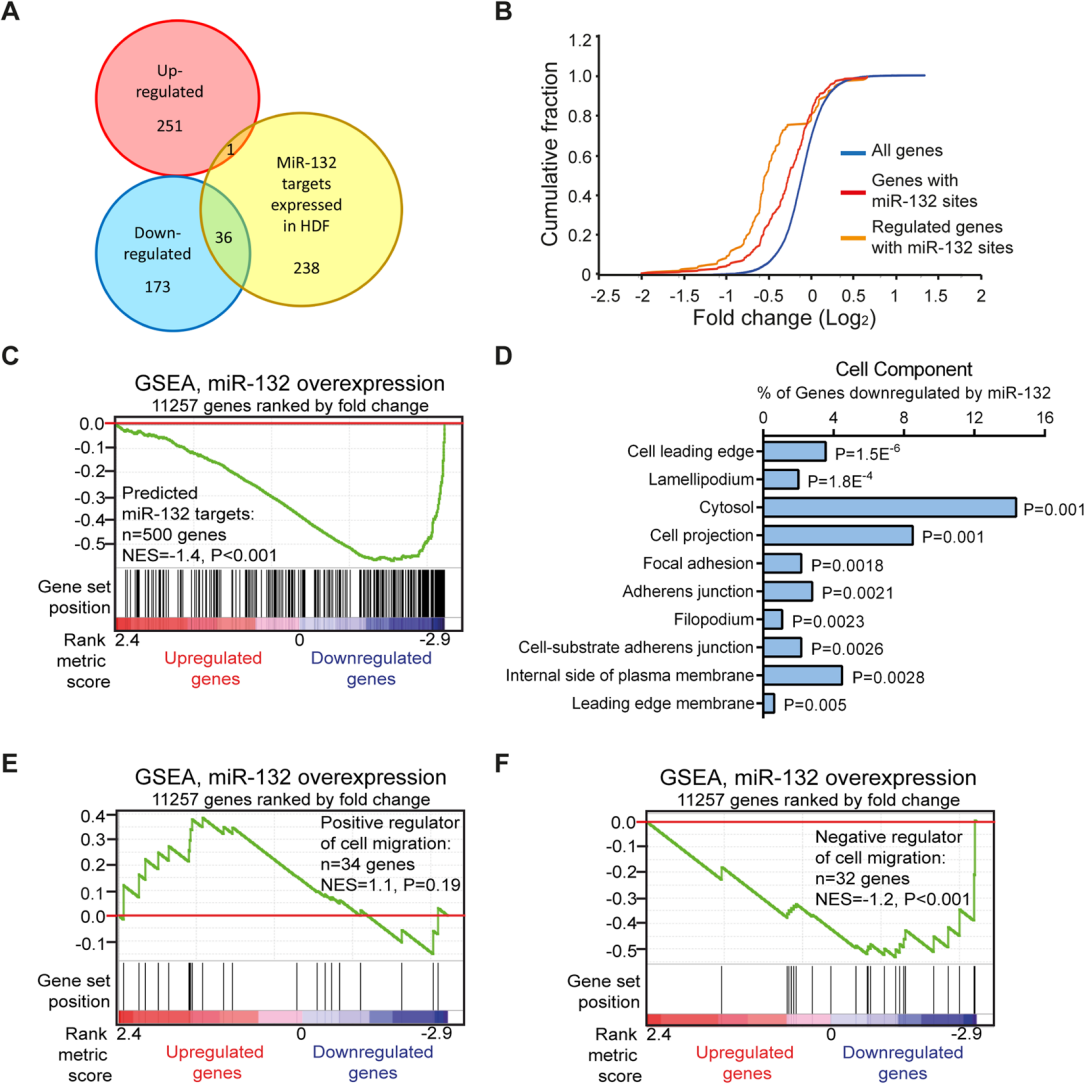
Transcriptome analysis of fibroblasts overexpressing miR-132. (**A**) Venn diagram depicting the number of genes significantly up- (red) or down-regulated (blue) by miR-132 (fold change ≥ 1.5, *P* <0.05) and TargetScan predicted miR-132 targets that expressed in HDFs (yellow). (**B**) Cumulative distribution plots of log_2_-transformed fold-changes for genes predicted to contain miR-132 binding sites (red), genes with miR-132 sites and regulated by miR-132 (orange) and all the genes detected by microarray (blue). (**C**) Genes in microarray data were ranked by fold change (miR-132 mimic / Ctrl mimic). GSEA evaluated enrichment within the profile data for the predicted target genes of miR-132. Vertical bars along the χ axis denote the positions of miR-132 target genes within the ranked list. NES, normalized enrichment score. (**D**) The top10 gene ontology cell component terms for the genes down-regulated by miR-132 in fibroblasts. *P*-values were determined by the Fisher’s exact test. GSEA evaluated enrichment within the microarray data for the genes reported to accelerate (**E**) or impair (**F**) cell migration.

To study the role of miR-132 in HDFs, we performed a global transcriptomic analysis of HDFs upon miR-132 overexpression (Supplementary Fig. S1) using Affymetrix arrays, which identified 461 genes significantly regulated (fold change ≥ 1.5 or ≤ −1.5, P < 0.05) by miR-132 (Fig. 2A, Supplementary Table S2). Out of the top 500 miR-132 targets predicted by the TargetScan algorithm^22^, 275 genes were found to be expressed in HDFs. Based on these 275 genes, we showed that 36 of 209 genes down-regulated by miR-132 carried potential binding sites for miR-132, whereas only 1 of 252 genes up-regulated by miR-132 was predicted to be targeted by miR-132 (Fig. 2A). We next analysed the correlation between the presence of miR-132–binding sites and the degree of regulation and found that mRNAs containing miR-132–binding sites displayed greater reduction in expression levels compared with other genes in this array (Fig. 2B). In line with this, gene set enrichment analysis (GSEA) showed that miR-132 target genes were significantly enriched among the genes down-regulated by miR-132, and a negative enrichment score curve was generated (P < 0.001) (Fig. 2C)^23^. These results suggest that the microarray analysis was specific and sensitive for detection of miR-132-mediated gene regulation.

Next, we performed gene ontological (GO) analysis to classify the genes regulated by miR-132 using the database for annotation, visualization and integrated discovery (DAVID) v6.7^24, 25^. We found that among the top 10 cell components enriched in the genes regulated by miR-132, most were related to cell motility, e.g. cell leading edge, lamellipodium, cell projection, focal adhesion, adherence junction and filopodium (Fig. 2D), which was further supported by GSEA showing that multiple migration-related processes were significantly (P < 0.001) enriched among the genes down-regulated by miR-132 (Supplementary Fig. S2). Using siRNA-screening approach, a group of genes have been previously identified to either negatively or positively regulate cell migration^26^. Interestingly, GSEA of microarray data showed that most positive regulators of cell migration were up-regulated by miR-132, whereas the expression of most negative regulators of cell migration was decreased by miR-132 in HDFs (Fig. 2E,F and Supplementary Fig. S3). Together, the results of the transcriptome analysis prompted us to further explore the potential role of miR-132 in motility of HDFs.

### MiR-132 promotes human dermal fibroblast migration

**Figure 3:**
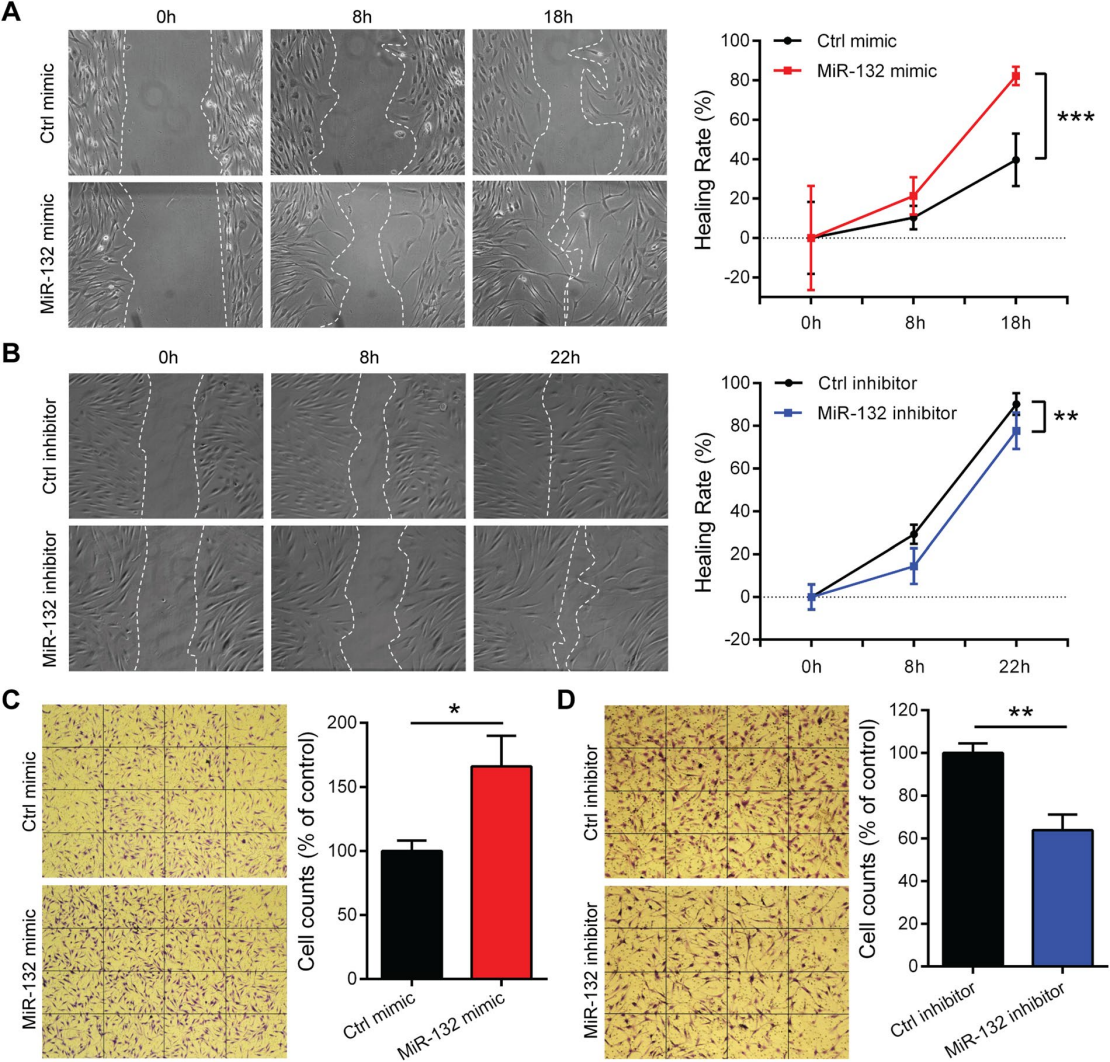
MiR-132 promotes fibroblast migration. Scratch assays were performed to assess the migration rate of HDFs transfected with 20 nM miR-132 mimics (**A**) or miR-132 inhibitors (**B**) for 48 hours. Photographs were taken at indicated time points after scratch injury. The healing rates were quantified by measuring the area of the injured region. **P < 0.01 and***P < 0.001 by Repeated Measures Two-way ANOVA. Transwell migration assay were performed to assess the motility of fibroblasts transfected with 20 nM miR-132 mimics (**C**) or miR-132 inhibitors (**D**). The number of fibroblasts passing through the membrane within 24 hours was counted. *P < 0.05 and **P < 0.01 by Student’s t-test. The data are presented as mean ± s.d.

To determine the role of miR-132 in fibroblast migration, we overexpressed or inhibited miR-132 by transfecting HDFs with miR-132 mimics or inhibitors, respectively (Supplementary Fig. S1). By scratch wound healing assay, we showed that the overexpression of miR-132 significantly promoted (P = 0.0006, Fig. 3A), whereas its inhibition significantly reduced the motility of HDFs (P = 0.006, Fig. 3B). Moreover, by using transwell migration assay, we found that miR-132 mimics accelerated (1.66-fold change, P = 0.011, Fig. 3C), while miR-132 inhibitors decreased (0.64-fold change, P = 0.0019, Fig. 3D) the migration of HDFs towards fetal bovine serum (FBS). Thus, we concluded that miR-132 promoted the motility of dermal fibroblasts.

### MiR-132 downregulates RASA1, which contributes to the pro-migratory function of miR-132 in fibroblasts

**Figure 4:**
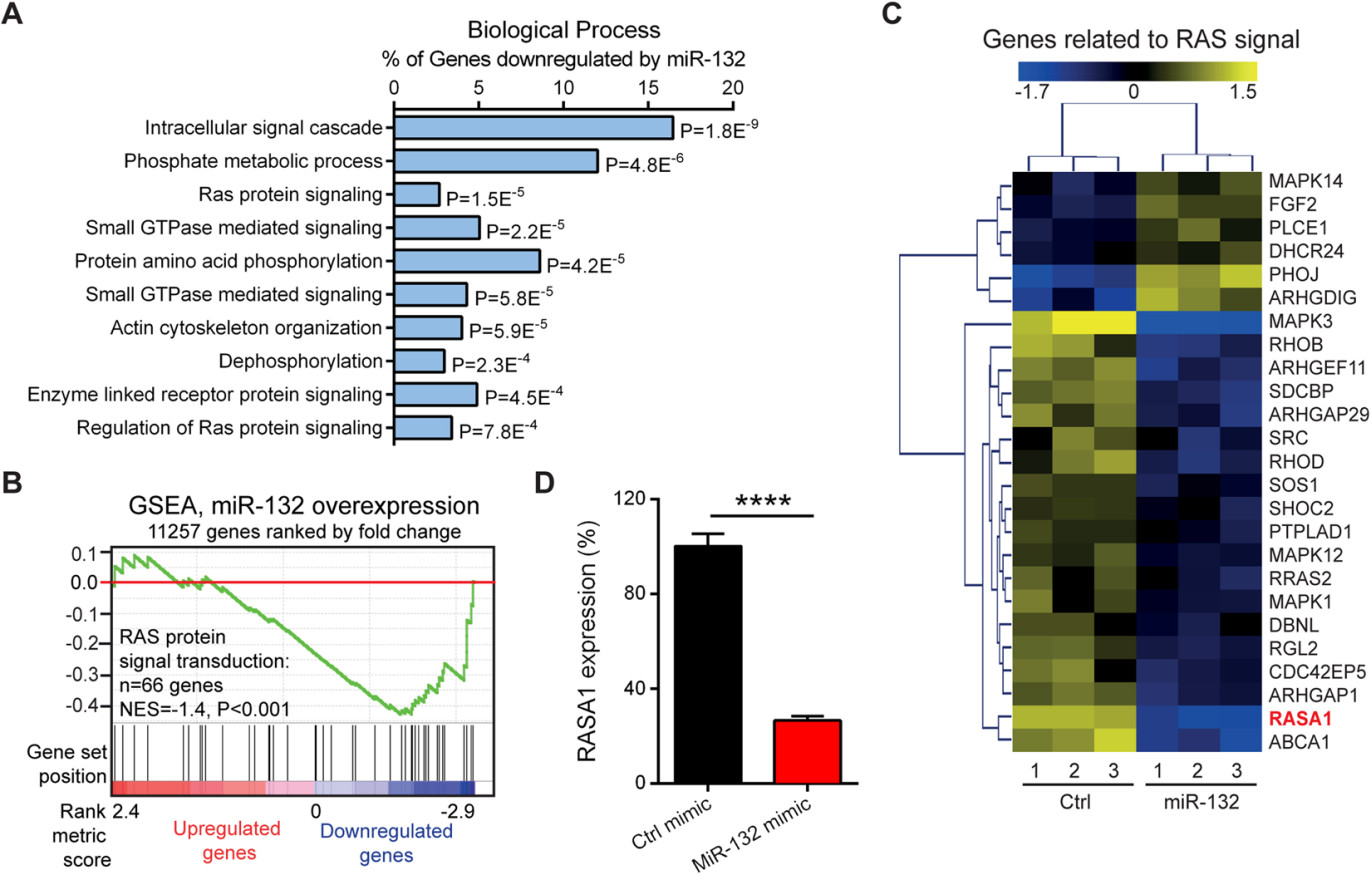
MiR-132 regulates RAS signalling pathway. (**A**) The top10 gene ontology biological process terms for the genes down-regulated by miR-132 in fibroblasts. *P*-values were determined by the Fisher’s exact test. (**B**) GSEA evaluated enrichment for the genes related to the RAS signalling within the microarray data. (**C**) The genes related to the RAS signalling were regulated by miR-132 in fibroblasts. Heat map illustrates levels of significantly changed genes (fold change ≥ 1.2, *P* < 0.05). Colour intensity is scaled within each row so that the highest expression value corresponds to bright yellow and the lowest to bright blue. (**D**) RASA1 was detected by qRT-PCR in HDFs transfected with 20 nM miR-132 mimics or control oligos (n = 3). The data are presented as mean ± s.d. ****P < 0.0001 by Student’s t test.

**Figure 5:**
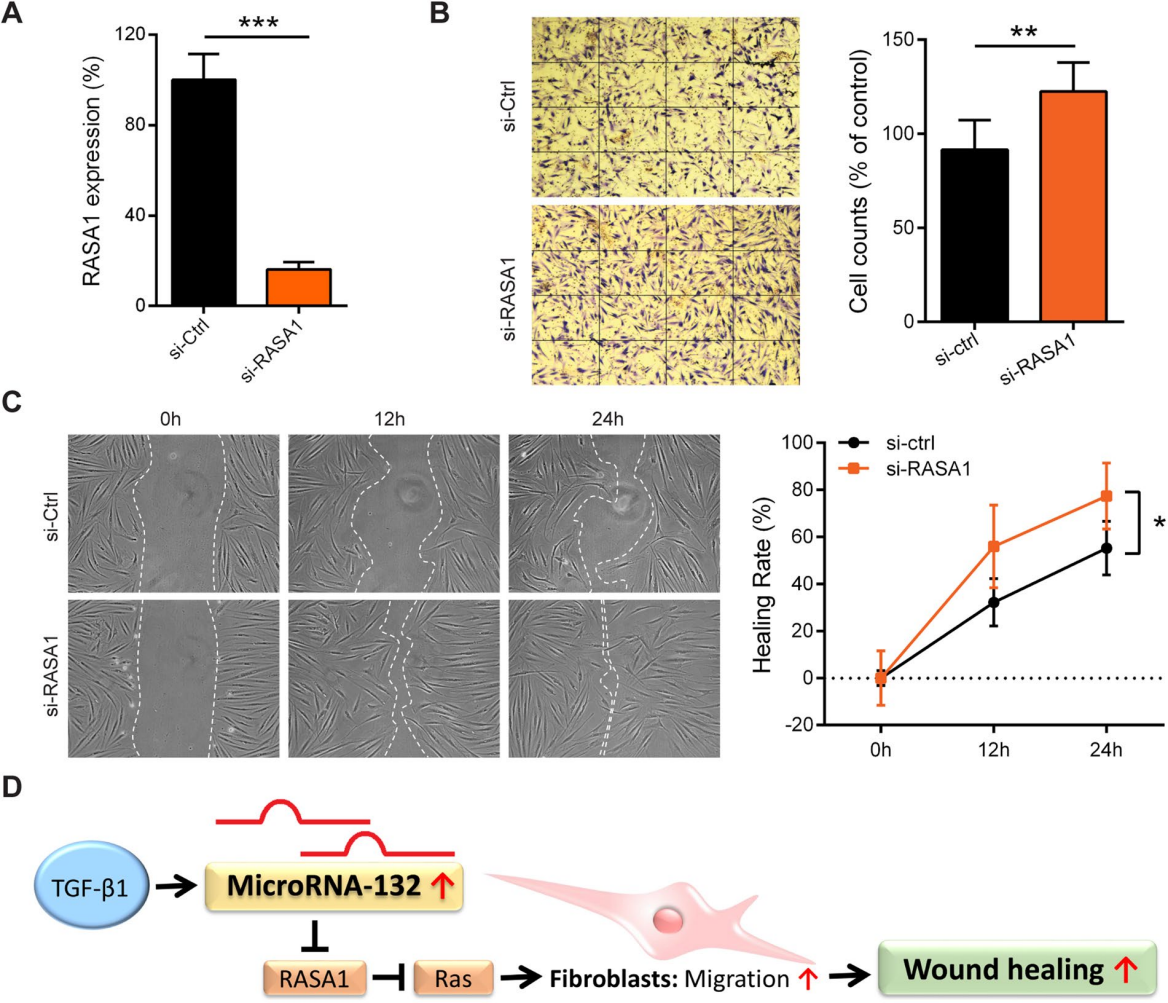
MiR-132 promotes migration by regulating RASA1. (**A**) RASA1 knockdown efficiency was analysed by qRT-PCR in HDFs transfected with 20 nM RASA1 specific siRNAs or control siRNAs (n = 3). (**B**) Transwell migration assay was performed in HDFs with silenced RASA1 expression (n = 3). **P < 0.01 and ***P < 0.001 by Student’s t test. (**C**) Scratch assay was performed in HDFs with silenced RASA1 expression. *P < 0.05 by Repeated Measures Two-way ANOVA. The data are presented as mean ± s.d. (**D**) Schematic summary of the role of miR-132 in dermal fibroblasts during skin wound healing.

The GO analysis and GSEA of microarray data highlighted that several biological processes related to the RAS and small GTPase signalling pathways, which are important for cell migration, growth, apoptosis and differentiation^27^, were regulated by miR-132 in HDFs (Fig. 4A,B). The genes related to the RAS signal (GO: 0007265) and significantly regulated (fold change ≥ 1.2, P < 0.05) by miR-132 in HDFs were listed in Fig. 4C. One of the top-down-regulated genes by miR-132 (fold change = −2.04, P = 7.4E-06, Supplementary Table S2) was RAS p21 protein activator 1 (RASA1), which was previously identified as a direct target of miR-132^28, 29^. We confirmed the miR-132-mediated downregulation of RASA1 in HDFs by qRT-PCR (0.27-fold change, P = 0.000024, Fig. 4D). Using siRNAs, we decreased the expression of RASA1 84% (Fig. 5A), which enhanced the motility of HDFs, shown by transwell migration assays (Fig. 5B) and scratch wound healing assays (Fig. 5C). Silencing of RASA1 expression phenocopied the miR-132 overexpression in HDFs, suggesting that RASA1 is an important target mediating the pro-migratory function of miR-132.

## Discussion

Our study reveals the important role of miR-132 in dermal fibroblasts during skin wound healing: it is upregulated by TGF-β1 and promotes fibroblast migration by regulating RASA1 expression (Fig. 5D). TGF-β is a pleiotropic growth factor playing important roles throughout all phases of wound healing. It regulates cell proliferation, migration, differentiation, extracellular matrix production and immune response^30^. Specific deletion of TGF-β receptor type II (TβRII) in fibroblasts severely impaired wound closure and delayed re-epithelialization *in vivo* ^31^. Together with our previous study^20^, we showed that TGF-β1 induced miR-132 expression in both epidermal keratinocytes and dermal fibroblasts. Upregulated miR-132 expression during skin wound healing may be due to the increased TGF-β1 production in the wound. Moreover, TGF-β signalling has been shown to be deficient in chronic wounds, e.g. fibroblasts derived from chronic wound-edge express reduced levels of TβRII and under-phosphorylated Smad2, a downstream signal of TβRII^32–35^. Thus, it would be interesting to further investigate whether the impaired TGF-β signalling leads to reduced miR-132 expression in chronic wounds.

In this study, we found that miR-132 enhanced migration of dermal fibroblasts. To explore the underlying molecular mechanism, we analysed the transcriptome of fibroblasts overexpressing miR-132, which identified RAS signaling as a major pathway regulated by miR-132 in HDFs. RAS are small GTPase, which can bind and hydrolyze guanosine triphosphate (GTP)^36^. RAS switches between an active (GTP-bound) and an inactive [guanosine diphophate (GDP)-bound] state^36^. Upon stimulation of tyrosine kinase / growth factor receptors, activated GTP-bound RAS acts through its effector proteins, e.g. RAF, PI3K, and trigger various signalling cascades, controlling cell migration, proliferation, differentiation and apoptosis^36^. Importantly, RAS signalling has been shown to play a crucial role in motility of fibroblasts and keratinocytes during skin wound healing^37^.

Among genes related to the RAS-signalling, we focused on RASA1, which was previously identified as a direct target of miR-132^28^, and we found that it was significantly down-regulated by miR-132 in dermal fibroblasts. RASA1 is one of the critical RAS GTPase activating proteins (RasGAP), which negatively regulates RAS activity by increasing the rate of GTP hydrolysis^38^. Moreover, RASA1 also functions as a signal transducer, independent of its GAP activity^38^. For instance, RASA1 has been shown to regulate directed movement of fibroblasts by interacting with p190 RhoGAP^39^. It also suppresses cell migration by competing with Rab21, a Rab GTPase, for binding to integrins and regulating receptor trafficking^40^. In line with this, we showed that silencing of RASA1 expression increased the motility of HDFs, which phenocopied the pro-migratory effects of miR-132, suggesting that the RASA1-RAS signal axis plays a key role in mediating the biological functions of miR-132 in fibroblasts.

We have previously shown that miR-132 facilitates inflammation-proliferation transition in epidermal keratinocytes^20^, and here we further demonstrate that miR-132 promotes dermal fibroblasts migration, both of which processes are impaired in chronic non-healing wounds (Fig. 5D). Thus, we propose that miR-132 holds great promise for treating chronic wounds.

## Methods

### Tissue samples

Healthy donors (Caucasian) were enrolled at the Unit of Dermatology and Venereology, Karolinska University Hospital (Stockholm, Sweden) (Supplementary Table S1). Two surgical wounds (3 mm in diameter, wound A and B) were created on the lower back area of each healthy volunteer and the skin excised during this procedure was collected as a control. For donor 1–5, the wound-edge tissue was excised using a 6 mm biopsy punch 1 day after injury from wound A and 7 days after injury from wound B for laser capture microdissection (LCM, Fig. 1A). For donor 6–10, the wound-edge tissue was excised 6 days after injury from both wound A and B for magnetic cell isolation.

### Laser capture microdissection (LCM)

Frozen tissue samples were embedded in Tissue-Tek (ThermoFisher Scientific), and 8-μm tissue sections were cut and stained with hematoxylin. LCM was performed with Leica LMD7000 (Leica).

### Magnetic cell separation

Fresh tissue samples were washed 2–3 times in PBS and incubated in 5 U/ml dispase (ThermoFisher Scientific) overnight at 4 °C. Epidermis was separated from dermis as described in ref. 41. Epidermis was diced using scissors and then digested in Trypsin/EDTA Solution (ThermoFisher Scientific) for 15 minutes at 37 °C, from which CD45- (mainly composed of keratinocytes) and CD45 + cells (leukocytes) were separated using CD45 Microbeads with MACS MS magnetic columns (Milteney Biotec). Dermis was incubated in the enzyme mix from the whole skin dissociation kit (Milteney Biotec) for 3 hours according to manufacturer’s instructions and further processed by Medicon tissue disruptor (BD Biosciences). Dermal cell suspension was sorted sequentially through CD90 (fibroblasts), CD14 (macrophages) and CD3 (T cells) Microbeads with MACS MS magnetic columns according to manufacturer’s instructions (Milteney Biotec). And the CD90- CD14- CD3- dermal cells were also collected.

### RNA extraction and qRT-PCR

Total RNA from human LCM tissues and from cells of wound tissues was extracted using the miRNeasy Mini kit (Qiagen) and that from cultured cells was extracted using TRIzol reagents (ThermoFisher Scientific).

Quantification of miRNAs by qRT-PCR was performed as previously described^20^. MiRNA expression levels were normalized between different samples on the basis of the values of RNU48 RNA. The mature miR-132 expression was quantified using the TaqMan miRNA assay (TM000457, ThermoFisher Scientific). The primary miR transcripts were quantified using the TaqMan Pri-miRNA assay (Hs03303111_pri, ThermoFisher Scientific).

In order to quantify mRNAs, total RNA was reverse transcribed using the RevertAid First Strand cDNA Synthesis Kit (ThermoFisher Scientific). RASA1 mRNAs were quantified by TaqMan gene expression assays (Integrated DNA Technologies). Target gene expression levels were normalized between samples to the internal control 18 S rRNA (forward: 5′-CGGCTACCACATCCAAGGAA-3′; reverse: 5′-GCTGGAATTACCGCGGCT-3′; probe: 5′-FAM-TGCTGGCACCAGACTTGCCCTC-TAMRA-3′).

### Cell isolation, culture and treatments

HDFs were isolated from adult human skin from routine abdominal reduction plastic surgery (n = 4). Briefly, full depth skin was enzymatic digested by 5 U dispase (ThermoFisher Scientific) overnight at 4 °C, and then epidermis and dermis were mechanically separated. The dermis was cut into small pieces and washed with PBS. After incubated at room temperature for 30 minutes, the dermis attached to plastic cell culture plate. DMEM medium supplemented with 10% heat inactivated FBS and 1% penicillin streptomycin (ThermoFisher Scientific) was then added into the culture plate, which was kept at 37 °C in 5% CO_2_. Fibroblasts grew out and the culture became confluent in approximately 2 weeks. The third to tenth passages of HDFs were used in this study.

HDFs were treated with TGF-β1 (10 ng/mL, Immuno tools) for 48 hours. TGF-β receptor inhibitor, SB431542 (15 μM, Tocris), or dimethylsulfoxide (DMSO) as control was applied 15 minutes before adding TGF-β1.

To study the functions of miR-132 in HDF, HDFs at 60–70% confluence were transfected with 20 nM mirVana^™^ miRNA mimic for has-miR-132–3p (miR-132 mimics) or miR mimic negative control 1 (Ctrl mimic) (ThermoFisher Scientific); 20 nM miR-132 miRCURY LNA Power inhibitor (miR-132 inhibitor) or negative control A (Ctrl inhibitor) (Exiqon) using Lipofectamine 2000 (ThermoFisher Scientific). The successful modulation of miR-132 expression level was confirmed by qRT-PCR (Supplementary Fig. S1). To study the function of RASA1, 20 nM Silencer select predesigned siRNA for RASA1 (si-RASA1 ID: s4353) or siRNA negative control number 1 (si-Ctrl) (ThermoFisher Scientific) were transfected into HDFs using Lipofectamine 2000 (ThermoFisher Scientific).

### Gene expression microarray

Expression profiling of HDFs transfected with 20 nM miR-132 mimics or Ctrl mimics for 48 hours (in triplicates) was performed using Affymetrix Genechip system at the microarray core facility of Karolinska Institute. In brief, total RNA was extracted using the miRNeasy Mini Kit and RNA quality and quantity were determined using Agilent 2100 Bioanalyzer and Nanodrop 1000. 100 ng of total RNA were used to prepare cDNA following the Affymetrix 3′IVT Express Kit labelling protocol. Standardized array processing procedures recommended by Affymetrix including hybridization, fluidics processing and scanning were used. Genes showing at least 1.5-fold regulation and P value less than 0.05 were considered to be differentially expressed. Gene ontology analysis was performed according to the Database for Annotation, Visualization and Integrated Discovery (DAVID v6.7)^24, 25^. Gene set enrichment analysis (GSEA) was performed using public software from Broad Institute^23^. The data discussed herein have been deposited in the NCBI’s Gene Expression Omnibus (GEO)^42^ (GEO GSE87436).

### Analysis of cell motility

For scratch assay, HDFs transfected with 20 nM miR-132 mimics or miR-132 inhibitors or si-RASA1 were grown to full confluence at collagen-coated 6-well plate and a scratch was made with a 10 μL pipette tip. The cells were incubated with DMEM medium with mitomycin C (10 μg/mL, Sigma-Aldrich) and photographed at the indicated time points. The wound areas were measured using Image J (National Institutes of Health). Healing rate = 100% - percentage of the initial wound area size.

Transwell migration assay was performed using the BD Chamber (BD Falcon). HDFs were transfected with 20 nM miR-132 mimics or miR-132 inhibitors or si-RASA1 for 48 hours. 1 × 10^5^ transfected cells in serum-free DMEM medium were placed into the upper chamber of the insert. Medium containing 10% FBS was added into the lower chamber. 24 hours later, the cells migrating through the membrane were stained with 0.1% crystal violet and counted under microscope.

### Statistics

Statistical significance was determined by two tailed Student’s t-test. Differences between groups were computed using two-way repeated-measures ANOVA. The *P*-values of enrichment of biological processes for the genes regulated by miR-132 were calculated by using Fisher’s exact test. *P*-value < 0.05 was considered to be statistically significant.

### Study approval

Written informed consent was obtained from all donors for the collection and use of clinical samples. The study was approved by the Stockholm Regional Ethics Committee (Stockholm, Sweden). The study was conducted according to the Declaration of Helsinki’s principles.

### Data availability

All data generated or analysed during this study are included in this published article (and its Supplementary Information files).

## Electronic supplementary material

Supplementary Information

## References

[1] Reinke JM, Sorg H (2012). Wound repair and regeneration. Eur Surg Res.

[2] Gottrup F (2004). A specialized wound-healing center concept: importance of a multidisciplinary department structure and surgical treatment facilities in the treatment of chronic wounds. Am J Surg.

[3] Frykberg RG, Banks J (2015). Challenges in the Treatment of Chronic Wounds. Adv Wound Care (New Rochelle).

[4] Gottrup F, Holstein P, Jorgensen B, Lohmann M, Karlsmar T (2001). A new concept of a multidisciplinary wound healing center and a national expert function of wound healing. Arch Surg.

[5] Richmond NA, Maderal AD, Vivas AC (2013). Evidence-based management of common chronic lower extremity ulcers. Dermatol Ther.

[6] Darby IA, Laverdet B, Bonte F, Desmouliere A (2014). Fibroblasts and myofibroblasts in wound healing. Clin Cosmet Investig Dermatol.

[7] Diegelmann RF, Evans MC (2004). Wound healing: an overview of acute, fibrotic and delayed healing. Front Biosci.

[8] Martin P, Nunan R (2015). Cellular and molecular mechanisms of repair in acute and chronic wound healing. Br J Dermatol.

[9] Harding KG, Moore K, Phillips TJ (2005). Wound chronicity and fibroblast senescence–implications for treatment. Int Wound J.

[10] Telgenhoff D, Shroot B (2005). Cellular senescence mechanisms in chronic wound healing. Cell Death Differ.

[11] Vande Berg JS, Rudolph R, Hollan C, Haywood-Reid PL (1998). Fibroblast senescence in pressure ulcers. Wound Repair Regen.

[12] Brem H (2008). Primary cultured fibroblasts derived from patients with chronic wounds: a methodology to produce human cell lines and test putative growth factor therapy such as GMCSF. J Transl Med.

[13] Brem H (2007). Molecular markers in patients with chronic wounds to guide surgical debridement. Mol Med.

[14] Khamaisi M (2016). PKCdelta inhibition normalizes the wound-healing capacity of diabetic human fibroblasts. J Clin Invest.

[15] Lerman OZ, Galiano RD, Armour M, Levine JP, Gurtner GC (2003). Cellular dysfunction in the diabetic fibroblast: impairment in migration, vascular endothelial growth factor production, and response to hypoxia. Am J Pathol.

[16] Loot MA (2002). Fibroblasts derived from chronic diabetic ulcers differ in their response to stimulation with EGF, IGF-I, bFGF and PDGF-AB compared to controls. Eur J Cell Biol.

[17] Cook H, Davies KJ, Harding KG, Thomas DW (2000). Defective extracellular matrix reorganization by chronic wound fibroblasts is associated with alterations in TIMP-1, TIMP-2, and MMP-2 activity. J Invest Dermatol.

[18] Wall SJ, Sampson MJ, Levell N, Murphy G (2003). Elevated matrix metalloproteinase-2 and -3 production from human diabetic dermal fibroblasts. Br J Dermatol.

[19] Jonas S, Izaurralde E (2015). Towards a molecular understanding of microRNA-mediated gene silencing. Nat Rev Genet.

[20] Li D (2015). MicroRNA-132 enhances transition from inflammation to proliferation during wound healing. J Clin Invest.

[21] Mi Q, Riviere B, Clermont G, Steed DL, Vodovotz Y (2007). Agent-based model of inflammation and wound healing: insights into diabetic foot ulcer pathology and the role of transforming growth factor-beta1. Wound Repair Regen.

[22] Agarwal, V., Bell, G. W., Nam, J. W. & Bartel, D. P. Predicting effective microRNA target sites in mammalian mRNAs. *Elife* **4**, doi:10.7554/eLife.05005 (2015). 10.7554/eLife.05005 PMC4532895 26267216

[23] Subramanian A (2005). Gene set enrichment analysis: a knowledge-based approach for interpreting genome-wide expression profiles. Proc Natl Acad Sci U S A.

[24] Huang da W, Sherman BT, Lempicki RA (2009). Systematic and integrative analysis of large gene lists using DAVID bioinformatics resources. Nat Protoc.

[25] Huang da W, Sherman BT, Lempicki RA (2009). Bioinformatics enrichment tools: paths toward the comprehensive functional analysis of large gene lists. Nucleic Acids Res.

[26] Simpson KJ (2008). Identification of genes that regulate epithelial cell migration using an siRNA screening approach. Nat Cell Biol.

[27] Kano Y, Cook JD, Lee JE, Ohh M (2016). New structural and functional insight into the regulation of Ras. Semin Cell Dev Biol.

[28] Anand S (2010). MicroRNA-132-mediated loss of p120RasGAP activates the endothelium to facilitate pathological angiogenesis. Nat Med.

[29] Hancock ML, Preitner N, Quan J, Flanagan JG (2014). MicroRNA-132 is enriched in developing axons, locally regulates Rasa1 mRNA, and promotes axon extension. J Neurosci.

[30] Lichtman MK, Otero-Vinas M, Falanga V (2016). Transforming growth factor beta (TGF-beta) isoforms in wound healing and fibrosis. Wound Repair Regen.

[31] Denton CP (2009). Inducible lineage-specific deletion of TbetaRII in fibroblasts defines a pivotal regulatory role during adult skin wound healing. J Invest Dermatol.

[32] Hasan A (1997). Dermal fibroblasts from venous ulcers are unresponsive to the action of transforming growth factor-beta 1. J Dermatol Sci.

[33] Jude EB, Blakytny R, Bulmer J, Boulton AJ, Ferguson MW (2002). Transforming growth factor-beta 1, 2, 3 and receptor type I and II in diabetic foot ulcers. Diabet Med.

[34] Kim BC (2003). Fibroblasts from chronic wounds show altered TGF-beta-signaling and decreased TGF-beta Type II receptor expression. J Cell Physiol.

[35] Pastar I (2010). Attenuation of the transforming growth factor beta-signaling pathway in chronic venous ulcers. Mol Med.

[36] Rajalingam K, Schreck R, Rapp UR, Albert S (2007). Ras oncogenes and their downstream targets. Biochim Biophys Acta.

[37] Ehrenreiter K (2005). Raf-1 regulates Rho signaling and cell migration. J Cell Biol.

[38] Pamonsinlapatham P (2009). p120-Ras GTPase activating protein (RasGAP): a multi-interacting protein in downstream signaling. Biochimie.

[39] Kulkarni SV, Gish G, van der Geer P, Henkemeyer M, Pawson T (2000). Role of p120 Ras-GAP in directed cell movement. J Cell Biol.

[40] Mai A (2011). Competitive binding of Rab21 and p120RasGAP to integrins regulates receptor traffic and migration. J Cell Biol.

[41] Cheuk S (2017). CD49a Expression Defines Tissue-Resident CD8+ T Cells Poised for Cytotoxic Function in Human Skin. Immunity.

[42] Edgar R, Domrachev M, Lash AE (2002). Gene Expression Omnibus: NCBI gene expression and hybridization array data repository. Nucleic Acids Res.

