# Supplementary material for: MicroRNA-132 promotes fibroblast migration via regulating RAS p21 protein activator 1 in skin wound healing
Source: Sci Rep. 2017 Aug 10;7:7797. doi: 10.1038/s41598-017-07513-0 (PMC5552762; doi:10.1038/s41598-017-07513-0)
Supplement: Supplementary file 1 — Supplementary Information [file 41598_2017_7513_MOESM1_ESM.pdf]

Supplementary Information for

MicroRNA-132 promotes fibroblast migration via regulating RAS p21 protein activator 1 in skin wound healing

Xi Li[1], Dongqing Li[1], Jakob D. Wikström[1,2], Andor Pivarcsi[1], Enikö Sonkoly[1,2], Mona Ståhle[1,2], Ning Xu Landén[1]*

[1]Unit of Dermatology and Venereology, Department of Medicine, Karolinska Institutet, Stockholm, Sweden. [2]Unit of Dermatology and Venereology, Karolinska University Hospital, Stockholm, Sweden

This PDF file includes:

Supplementary Figures 1 – 3

Supplementary Tables 1 – 2

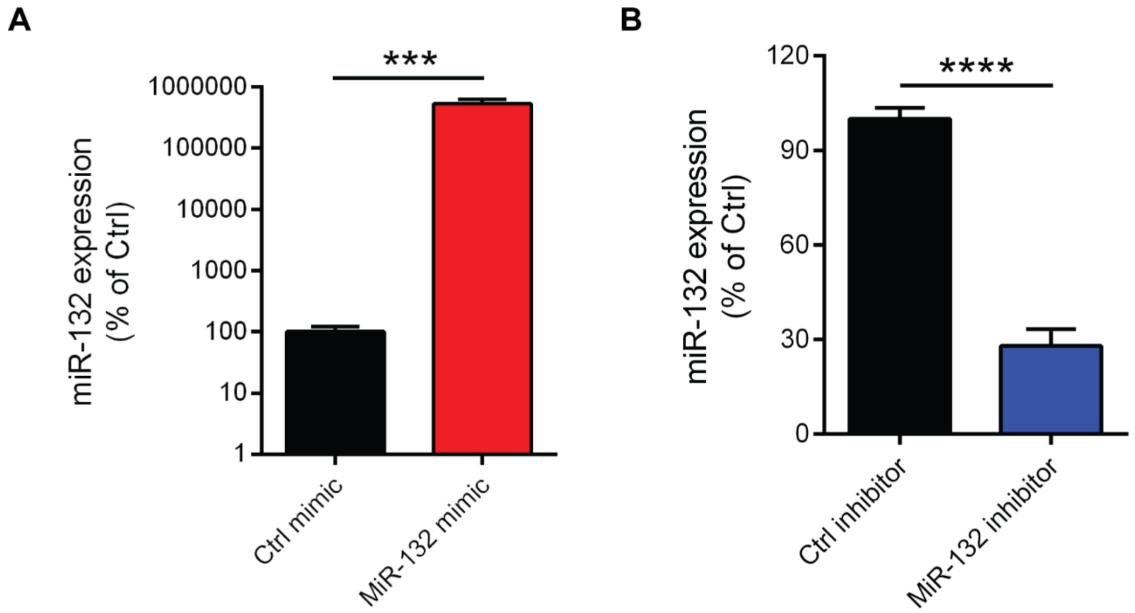

Supplementary Figure S1: Modulation of miR-132 levels in human dermal fibroblasts. QRT-PCR analysis of miR-132 expression in HDFs transfected with **(A)** 20nM miR-132 mimics or miRNA control mimics for 48 hours; **(B)** in HDFs transfected with 20nM miR-132 inhibitors or miRNA control inhibitors for 48 hours. The data are presented as mean ± s.d. ****P< 0.0001 by Student's t-test.

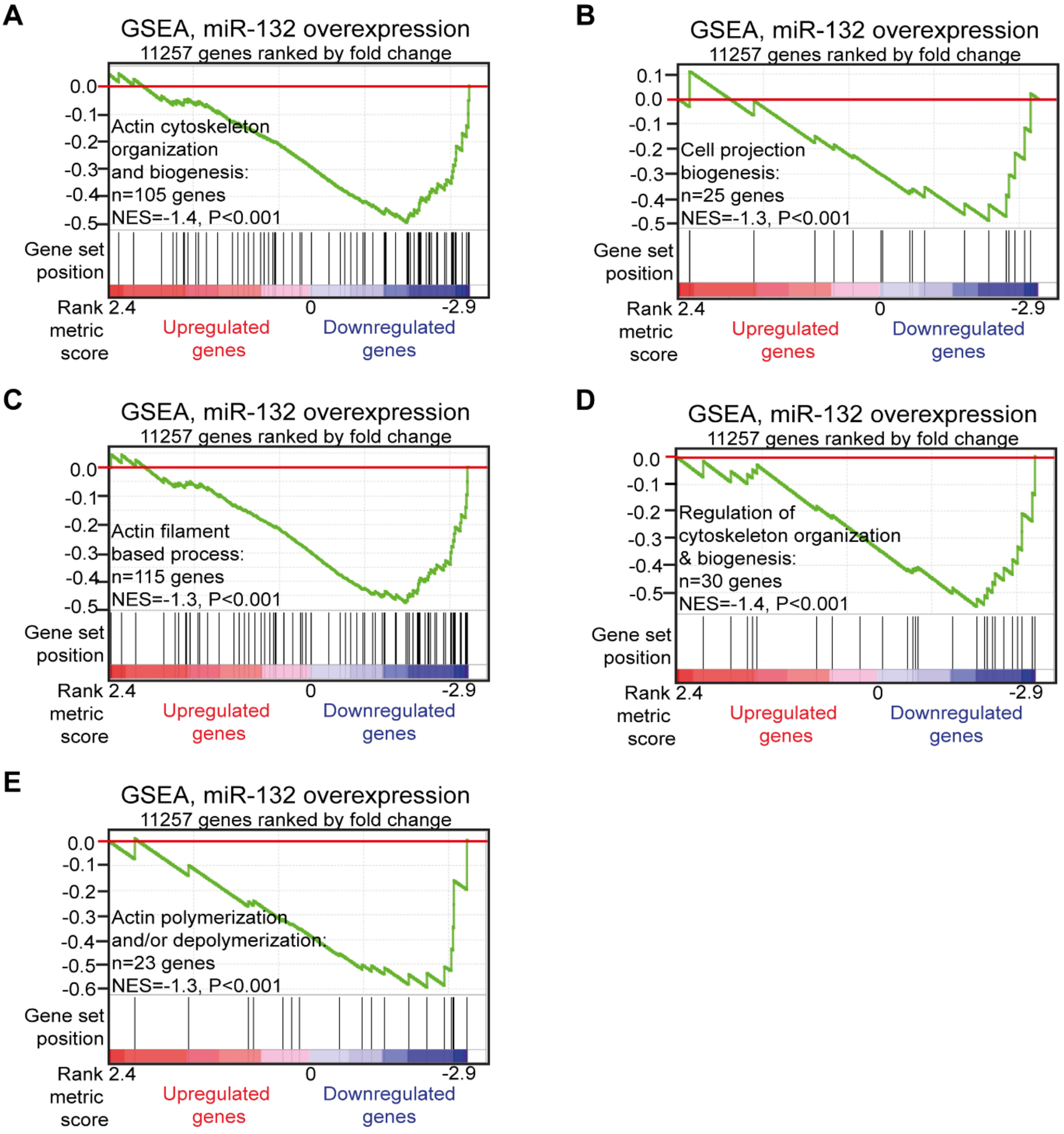

Supplementary Figure S2: GSEA evaluated enrichment for the genes related to cell migration in the microarray data. Genes in the microarray data of HDFs with miR-132 overexpression were ranked by fold change (miR-132 mimic / Ctrl mimic). GSEA evaluated enrichment within the profile data for the genes related to **(A)** actin cytoskeleton organization, **(B)** cell projection biogenesis, **(C)** actin filament based process, **(D)** regulation of cytoskeleton organization and biogenesis and **(E)** actin polymerization and/or depolymerization.

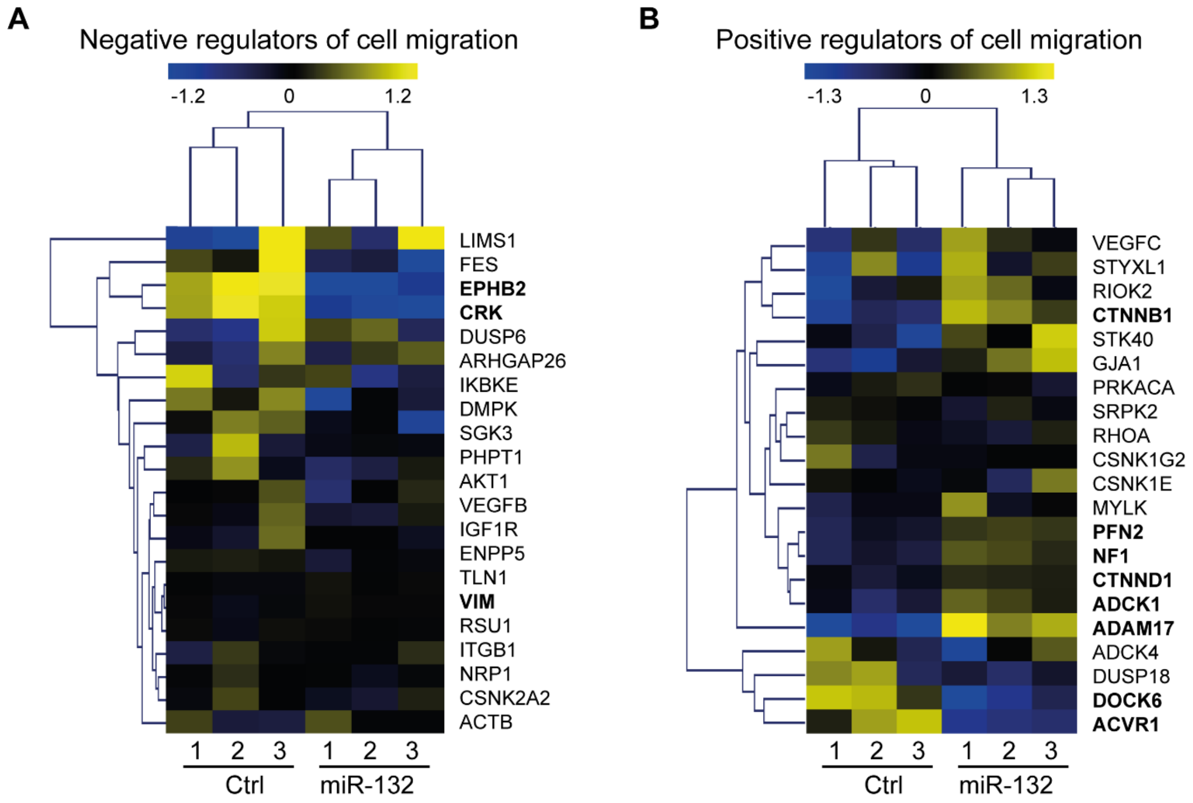

Supplementary Figure S3: The expression of cell migration regulators in human dermal fibroblast overexpressing miR-132. Using siRNA-screening approach, a group of genes have been previously identified to either negatively or positively regulate cell migration. Heat map illustrates the expression of these genes in the fibroblast overexpressing miR-132. Colour intensity is scaled within each row so that the highest expression value corresponds to bright yellow and the lowest to bright blue. The names of the genes significantly (P <0.05) changed by miR-132 overexpression were highlighted with bold font.

Supplementary Table S1: Information of healthy donors

Donor	Gender	Age (y)	Surgical wounds *in vivo*				Analysis
			Day 0	Day 1	Day 6	Day 7	
1	Male	33	√	√		√	LCM
2	Male	34	√	√		√	LCM
3	Female	58	√	√		√	LCM
4	Female	53	√	√		√	LCM
5	Male	23	√	√		√	LCM
6 (V1)	Male	27			√		MACS
7 (V2)	Male	33			√		MACS
8 (V3)	Female	36			√		MACS
9 (V4)	Male	25			√		MACS
10 (V5)	Female	38			√		MACS

Supplementary table 2: miR-132 regulated transcripts in human primary fibroblasts

The presence of miR-132 target binding sites was predicted by TargetScan.

Pre-miR-Ctrl (Log2)			Pre-miR-132 (Log2)			Average		Fold Change (miR-132 vs Ctrl)	p-value	Gene Assignment			Predicted miR-132 binding sites
Sample 1	Sample 2	Sample 3	Sample 1	Sample 2	Sample 3	Pre-miR-Ctrl	Pre-miR-132			Public id	Symbol	Name	
6,45	6,45	6,5	4,8	4,2	4,44	88,4547	22,6477	-3,9057	2,42532E-05	NM_144601	CMTM3	CKLF-like MARVEL transmembrane domain containing 3	Yes
6,59	6,58	6,51	4,66	4,83	4,65	94,3817	26,277	-3,5918	4,09223E-06	ENST00000300571	GPRC5B	G protein-coupled receptor, family C, group 5, member B	Yes
7,45	7,4	7,45	5,63	5,57	5,76	172,8677	50,4063	-3,4295	1,64384E-06	ENST00000233813	IGFBP5	insulin-like growth factor binding protein 5	
8,68	8,54	8,66	7,26	6,98	7,11	395,622	139,2187	-2,8417	5,48638E-05	NM_003107	SOX4	SRY (sex determining region Y)-box 4	Yes
6,1	6,09	5,83	4,44	4,22	4,8	64,5333	22,7333	-2,8387	0,000875614	ENST00000424383	ELFN1	extracellular leucine-rich repeat and fibronectin type III domain co	
7,9	8,06	8,07	6,3	6,63	6,67	258,1513	93,222	-2,7692	0,000166968	NM_001040056	MAPK3	mitogen-activated protein kinase 3	Yes
6,83	7,36	7,36	5,46	5,92	5,82	147,4433	53,686	-2,7464	0,005922346	ENST00000339732	GALNTL2	UDP-N-acetyl-alpha-D-galactosamine:polypeptide N-acetylgalacto	
6,74	7,2	7,12	5,56	5,86	5,51	131,0087	50,276	-2,6058	0,003313589	NM_001928	CFD	complement factor D (adipsin)	
6,8	7,27	7,31	5,59	5,81	6,09	141,4853	57,4637	-2,4622	0,00651416	ENST00000296130	CLEC3B	C-type lectin domain family 3, member B	
6,97	6,77	6,92	5,51	5,56	5,76	118,5327	48,9797	-2,42	0,000230226	NM_006923	SDF2	stromal cell-derived factor 2	Yes
7,48	7,53	7,44	6,49	5,94	6,19	178,9983	74,762	-2,3942	0,000301499	NM_015497	TMEM87A	transmembrane protein 87A	Yes
5,91	5,49	5,36	4,14	4,44	4,47	48,7137	20,4993	-2,3764	0,009248072	NM_005144	HR	hairless homolog (mouse)	
6,4	6,3	6,6	4,92	5,47	5,34	86,7493	38,3673	-2,261	0,002088776	NM_002579	PALM	paralemmin	
5,94	5,81	5,77	4,63	4,67	4,74	57,355	25,647	-2,2363	0,000121978	NM_001135642	INPP5K	inositol polyphosphate-5-phosphatase K	
5,67	5,84	5,47	4,38	4,73	4,39	50,84	22,775	-2,2323	0,002579144	NM_004548	NDUFB10	NADH dehydrogenase (ubiquinone) 1 beta subcomplex, 10, 22kD	Yes
6,05	6,24	6,53	4,93	5,35	5,22	78,084	36,1803	-2,1582	0,007018926	ENST00000296861	TNFRSF21	tumor necrosis factor receptor superfamily, member 21	
5,97	6,67	6,2	5,42	4,93	5,21	79,343	36,7707	-2,1578	0,025141514	ENST00000426925	SH3BP5	SH3-domain binding protein 5 (BTK-associated)	
5,45	5,98	6,11	4,86	4,75	4,7	58,6343	27,314	-2,1467	0,01531336	NM_002053	GBP1	guanylate binding protein 1, interferon-inducible	
7,74	8,04	7,64	6,69	6,73	6,77	225,482	106,18	-2,1236	0,003533214	ENST00000222271	COMP	cartilage oligomeric matrix protein	
5,98	6,29	5,93	5,03	4,95	5,01	67,4457	31,935	-2,112	0,002889349	NM_020422	TMEM159	transmembrane protein 159	
5,64	6,01	6,32	4,87	4,74	5,17	64,735	30,656	-2,1117	0,020040789	NM_017680	ASPN	asporin	
8,87	8,41	8,17	7,52	7,41	7,4	365,347	174,1717	-2,0976	0,02349608	NM_014424	HSPB7	heat shock 27kDa protein family, member 7 (cardiovascular)	
6,58	6,73	6,4	5,59	5,34	5,58	95,424	45,5023	-2,0971	0,001779847	BC104183	KIAA1644	KIAA1644	
5,72	5,35	5,69	4,15	4,9	4,47	48,3737	23,2573	-2,0799	0,008439	ENST00000394521	CAPS	calcyphosine	
8,86	9,05	8,88	7,9	7,91	7,82	488,614	235,1153	-2,0782	0,000285871	NM_001199214	STMN2	stathmin-like 2	
8,31	8,12	8,28	7,34	7,07	7,15	302,1343	146,1353	-2,0675	0,000440635	NM_003588	CUL4B	cullin 4B	
6,47	6,57	6,55	5,55	5,53	5,41	92,4527	45,1917	-2,0458	3,69008E-05	NM_001184819	GNL3L	guanine nucleotide binding protein-like 3 (nucleolar)-like	
8,17	8,17	8,14	7,22	7,08	7,09	286,0393	140,2077	-2,0401	0,000007386	NM_002890	RASA1	RAS p21 protein activator (GTPase activating protein) 1	Yes
6,14	5,86	5,8	4,42	5,26	4,95	61,4393	30,212	-2,0336	0,009642088	NR_024084	SAP30L	SAP30-like	Yes
6,99	7,03	6,83	6,09	5,84	5,86	123,8593	61,161	-2,0251	0,000544213	NM_001199159	PI16	peptidase inhibitor 16	
7,24	6,82	7,11	6,15	5,94	6,05	134,098	66,2207	-2,025	0,004181626	NM_003155	STC1	stanniocalcin 1	
7,98	7,97	7,9	6,86	7,03	6,91	247,3547	122,3703	-2,0214	0,00003316	NM_015975	TAF9B	TAF9B RNA polymerase II, TATA box binding protein (TBP)-asso	
7,35	7,27	7,22	6,17	6,41	6,22	155,5243	77,1943	-2,0147	0,000164812	ENST00000368911	CDK19	cyclin-dependent kinase 19	Yes
8,13	8,19	8,13	7,24	7,15	7,09	284,1047	143,1437	-1,9848	0,000017994	ENST00000373610	TXLNA	taxilin alpha	
7,1	6,98	7,12	6,2	6,07	5,98	134,1757	67,9393	-1,9749	0,000190753	NM_173470	MMGT1	membrane magnesium transporter 1	
6,17	5,71	5,81	4,8	4,82	5,17	60,151	30,702	-1,9592	0,011044444	NM_144653	NACC2	NACC family member 2, BEN and BTB (POZ) domain containing	Yes

Pre-miR-Ctrl (Log2)			Pre-miR-132 (Log2)			Average		Fold Change (miR-132 vs Ctrl)	p-value	Gene Assignment			Predicted miR-132 binding sites
Sample 1	Sample 2	Sample 3	Sample 1	Sample 2	Sample 3	Pre-miR-Ctrl	Pre-miR-132			Public id	Symbol	Name	
8,65	8,72	8,64	7,84	7,61	7,65	407,4393	208,447	-1,9546	9,62638E-05	NM_018490	LGR4	leucine-rich repeat containing G protein-coupled receptor 4	
6,58	6,76	6,61	5,72	5,61	5,81	100,578	52,551	-1,9139	0,000424867	NM_001031683	IFIT3	interferon-induced protein with tetratricopeptide repeats 3	
5,63	5,88	5,68	4,65	4,85	4,89	53,2273	27,866	-1,9101	0,001370358	NM_080881	DBN1	drebrin 1	
6,86	6,97	6,91	5,95	6,03	5,96	120,5957	63,1383	-1,91	3,74811E-05	NR_049789	PLEKHB2	pleckstrin homology domain containing, family B (evectins) memb	
7,05	7,22	7,4	6,25	6,41	6,23	150,1657	78,7353	-1,9072	0,002883503	NM_005100	AKAP12	A kinase (PRKA) anchor protein 12	
5,35	5,09	5,31	4,63	4,04	4,27	38,1723	20,168	-1,8927	0,004941776	ENST00000254801	IGJ	immunoglobulin J polypeptide, linker protein for immunoglobulin a	
5,77	5,34	5,75	4,91	4,77	4,42	49,63	26,252	-1,8905	0,011094482	NM_198472	C10orf125	chromosome 10 open reading frame 125	
6,6	6,41	6,85	5,8	5,86	5,45	99,134	52,503	-1,8882	0,009161725	NR_003246	GOLGA6L5	golgin A6 family-like 5 (pseudogene)	
5,76	5,93	6,1	4,9	4,98	5,17	61,2517	32,4727	-1,8863	0,003185213	NM_001033723	ZNF704	zinc finger protein 704	
8,48	8,7	8,6	7,6	7,74	7,7	386,9833	205,244	-1,8855	0,000538109	NM_058229	FBXO32	F-box protein 32	
8,07	8,26	8,17	7,34	7,24	7,18	287,7657	152,731	-1,8841	0,000355181	ENST00000300574	CRK	v-crk sarcoma virus CT10 oncogene homolog (avian)	Yes
5,85	5,48	5,8	4,79	5,01	4,59	52,6757	27,9907	-1,8819	0,006264542	NM_014586	HUNK	hormonally up-regulated Neu-associated kinase	
7,31	7,23	7,17	6,26	6,37	6,35	150,9377	80,3073	-1,8795	0,00010918	NM_203351	MAP3K3	mitogen-activated protein kinase kinase kinase 3	Yes
6,99	7,04	7,19	6,18	6,29	6,02	134,911	71,8823	-1,8768	0,000792784	NM_005502	ABCA1	ATP-binding cassette, sub-family A (ABC1), member 1	
6,46	6,38	6,57	5,59	5,34	5,75	88,777	47,4963	-1,8691	0,001309557	ENST00000560869	TNFAIP2	tumor necrosis factor, alpha-induced protein 2	
8,32	8,24	8,22	7,18	7,37	7,57	306,693	166,8163	-1,8385	0,000657671	ENST00000412716	DAZAP2	DAZ associated protein 2	Yes
7,25	7,31	7,27	6,47	6,57	6,12	155,0817	84,4027	-1,8374	0,000856024	NM_003632	CNTNAP1	contactin associated protein 1	
6,62	6,79	6,43	5,85	5,7	5,69	98,4147	53,763	-1,8305	0,003658216	NM_033339	CASP7	caspase 7, apoptosis-related cysteine peptidase	Yes
7,35	7,18	7,27	6,42	6,45	6,32	154,1653	84,3157	-1,8284	0,000255953	NM_006832	FERMT2	fermitin family member 2	
5,85	6,31	5,73	5	5,3	5,03	63,3657	34,6897	-1,8266	0,027284687	NM_024825	PODNL1	podocan-like 1	
6,62	6,88	6,79	5,79	5,95	5,96	108,935	59,8	-1,8217	0,001288984	NM_001242532	MFSD11	major facilitator superfamily domain containing 11	Yes
5,62	5,99	6,05	4,68	5,04	5,32	59,665	32,827	-1,8176	0,01621756	ENST00000443533	DDAH2	dimethylarginine dimethylaminohydrolase 2	
8,42	8,62	8,52	7,56	7,69	7,73	367,6807	202,504	-1,8157	0,000537622	NM_138420	AHNAK2	AHNAK nucleoprotein 2	
8,1	8,45	8,29	7,39	7,31	7,57	312,3587	172,144	-1,8145	0,004061307	NM_133373	PLCD3	phospholipase C, delta 3	
7,26	7,42	7,37	6,65	6,35	6,46	163,3177	90,0113	-1,8144	0,00066536	NM_003786	ABCC3	ATP-binding cassette, sub-family C (CFTR/MRP), member 3	
5,16	5,32	5,41	4,47	4,26	4,58	39,406	21,7467	-1,812	0,001851113	NM_015983	UBE2D4	ubiquitin-conjugating enzyme E2D 4 (putative)	
9,53	9,52	9,59	8,69	8,66	8,73	748,0543	414,038	-1,8067	1,28691E-05	NM_001017424	KCNK2	potassium channel, subfamily K, member 2	Yes
6,88	6,49	6,87	6,26	6	5,33	108,2127	60,2877	-1,7949	0,027127837	NM_018427	RRN3	RRN3 RNA polymerase I transcription factor homolog (S. cerevisi	
6,36	6,66	6,35	5,73	5,71	5,41	88,2787	49,3133	-1,7902	0,0058512	ENST00000544228	MTMR1	myotubularin related protein 1	
6,25	6,39	6,7	5,47	5,89	5,47	87,9807	49,3167	-1,784	0,016247056	NM_198827	GPR133	G protein-coupled receptor 133	
6,8	7,05	7,17	5,91	6,3	6,3	129,317	72,5717	-1,7819	0,007582937	ENST00000405308	MDK	midkine (neurite growth-promoting factor 2)	
5,75	5,94	6,02	4,89	5,37	4,92	60,0343	33,76	-1,7783	0,00633843	ENST00000422725	C1orf233	chromosome 1 open reading frame 233	
9,56	9,54	9,47	8,46	8,79	8,81	736,1453	414,5347	-1,7758	0,000709941	NM_001136191	KANK2	KN motif and ankyrin repeat domains 2	
6,41	6,11	6,34	5,35	5,6	5,46	78,3717	44,4353	-1,7637	0,003034201	NM_173505	ANKRD29	ankyrin repeat domain 29	Yes
7,79	7,83	7,81	6,97	7,01	7,03	224,4257	128,3153	-1,749	2,25208E-06	ENST00000322213	SMC1A	structural maintenance of chromosomes 1A	
7,59	7,69	7,38	6,81	6,61	6,85	188,5813	108,4157	-1,7394	0,003422491	NM_016097	IER3IP1	immediate early response 3 interacting protein 1	
7,61	7,36	7,49	6,72	6,74	6,62	179,803	103,557	-1,7363	0,001231308	NM_001955	EDN1	endothelin 1	
5,73	5,91	6,06	5,15	5,14	5,04	59,9743	34,5557	-1,7356	0,003217345	NM_152345	ANKRD13B	ankyrin repeat domain 13B	
7,57	7,44	7,44	6,65	6,74	6,68	179,103	103,285	-1,7341	0,00019508	NM_025250	TTYH3	tweety homolog 3 (Drosophila)	
8,12	8,24	8,34	7,33	7,55	7,44	301,524	173,982	-1,7331	0,001128768	NM_001204502	FLT3LG	fms-related tyrosine kinase 3 ligand	

Pre-miR-Ctrl (Log2)			Pre-miR-132 (Log2)			Average		Fold Change (miR-132 vs Ctrl)	p-value	Gene Assignment			Predicted miR-132 binding sites
Sample 1	Sample 2	Sample 3	Sample 1	Sample 2	Sample 3	Pre-miR-Ctrl	Pre-miR-132			Public id	Symbol	Name	
7,43	7,59	7,52	6,71	6,69	6,77	182,888	105,6927	-1,7304	0,000226029	NM_016626	MEX3C	mex-3 homolog C (C. elegans)	
5,77	5,69	5,98	4,81	5,11	5,14	56,4377	32,6157	-1,7304	0,004521578	NM_001164446	C6orf132	chromosome 6 open reading frame 132	
5,51	5,88	5,97	4,88	5,06	5,08	55,715	32,21	-1,7297	0,011915307	NM_001204286	MUC1	mucin 1, cell surface associated	
5,73	5,56	5,73	4,91	5,08	4,64	51,1097	29,6077	-1,7262	0,002680139	NM_058164	OLFM2	olfactomedin 2	
7,97	8,26	8,22	7,31	7,31	7,48	285,153	165,2977	-1,7251	0,00298215	NM_003004	SECTM1	secreted and transmembrane 1	
8,84	8,82	8,85	8,16	8,06	7,93	457,2123	265,5907	-1,7215	0,000105636	NM_001042537	SLC9A6	solute carrier family 9, subfamily A (NHE6, cation proton antiporte	
6,39	6,05	6,34	5,44	5,59	5,42	77,0433	44,7977	-1,7198	0,004851082	NM_001161766	AHCY	adenosylhomocysteinase	Yes
6,39	6,28	6,35	5,39	5,71	5,56	81,0483	47,152	-1,7189	0,000637516	ENST00000259392	SLC31A2	solute carrier family 31 (copper transporters), member 2	
8,01	7,98	7,72	7,04	7,17	7,18	240,3653	140,205	-1,7144	0,002922825	NM_004750	CRLF1	cytokine receptor-like factor 1	
5,58	6,03	6,02	4,75	5,02	5,48	59,3577	34,6627	-1,7124	0,03379438	ENST00000443533	DDAH2	dimethylarginine dimethylaminohydrolase 2	
6,8	6,5	6,89	5,89	6,05	5,95	106,8477	62,4597	-1,7107	0,006884507	NM_004102	FABP3	fatty acid binding protein 3, muscle and heart (mammary-derived g	
6,23	6,13	6,46	5,36	5,6	5,55	77,7103	45,4747	-1,7089	0,005183594	NM_022783	DEPTOR	DEP domain containing MTOR-interacting protein	
6,27	6,12	6,25	5,41	5,21	5,67	74,2773	43,482	-1,7082	0,002787044	NM_006598	SLC12A7	solute carrier family 12 (potassium/chloride transporters), member	
7,57	7,61	7,64	6,65	6,99	6,85	194,9487	114,301	-1,7056	0,000597265	NM_138383	MTSS1L	metastasis suppressor 1-like	
6,49	6,16	6,06	5,34	5,35	5,73	76,036	44,7887	-1,6977	0,018832336	NM_006989	RASA4	RAS p21 protein activator 4	
5,22	5,12	5,3	4,33	4,72	4,27	37,148	21,92	-1,6947	0,004236818	NM_052880	PIK3IP1	phosphoinositide-3-kinase interacting protein 1	
5,61	5,85	5,73	4,9	4,91	5,1	53,1987	31,4063	-1,6939	0,001754079	NM_017449	EPHB2	EPH receptor B2	
5,99	6,32	5,92	5,26	5,66	4,98	67,9997	40,147	-1,6938	0,027239791	NM_032515	BOK	BCL2-related ovarian killer	
7,53	7,36	7,32	6,73	6,45	6,74	169,6293	100,157	-1,6936	0,002253156	NM_134268	CYGB	cytoglobin	
7,73	7,72	7,72	7,03	6,78	7,08	211,328	125,2947	-1,6866	0,000389617	ENST00000258198	DYNC1LI2	dynein, cytoplasmic 1, light intermediate chain 2	
5,83	5,95	5,78	4,69	5,44	5,09	57,8847	34,428	-1,6813	0,012840257	NM_002015	FOXO1	forkhead box O1	
6,29	6,27	6,62	5,54	5,76	5,66	84,5937	50,4273	-1,6775	0,009164714	ENST00000240123	SORBS3	sorbin and SH3 domain containing 3	
8,22	8,45	8,4	7,34	7,8	7,67	328,5573	196,1783	-1,6748	0,005094719	NM_005512	LRRC32	leucine rich repeat containing 32	
6,62	6,49	6,68	5,8	5,88	5,9	96,927	58,107	-1,6681	0,000581778	NM_014992	DAAM1	dishevelled associated activator of morphogenesis 1	
7,55	7,76	7,99	6,94	7	7,18	219,4673	131,9317	-1,6635	0,012885449	NM_001077195	ZNF436	zinc finger protein 436	
7,43	7,23	7,42	6,73	6,54	6,61	164,608	98,9627	-1,6633	0,001321967	ENST00000366862	FBXO28	F-box protein 28	Yes
8,69	8,6	8,69	7,96	7,97	7,85	404,675	243,484	-1,662	0,000105139	NM_015137	EFR3A	EFR3 homolog A (S. cerevisiae)	
6,33	6,31	6,3	5,51	5,49	5,73	79,5277	47,8627	-1,6616	0,000284384	NM_017857	SSH3	slingshot homolog 3 (Drosophila)	
6,87	6,85	6,63	6,3	6,03	5,79	110,458	66,4893	-1,6613	0,007776731	NM_022900	CASD1	CAS1 domain containing 1	
8,54	8,71	8,56	7,73	7,97	7,92	389,4653	235,0763	-1,6568	0,001192754	NM_007173	PRSS23	protease, serine, 23	
6,64	6,51	6,42	5,99	5,7	5,69	92,1663	55,7223	-1,654	0,003022555	ENST00000494426	CLIC3	chloride intracellular channel 3	
5,7	6,02	6,09	5,38	5,23	5,04	61,6657	37,358	-1,6507	0,011795758	ENST00000242576	UNG	uracil-DNA glycosylase	
8,8	8,86	8,89	8,05	8,03	8,29	461,595	279,801	-1,6497	0,000619355	NR_028349	LOC100287834	uncharacterized LOC100287834	
5,32	4,8	5,02	4,24	4,57	4,18	33,4173	20,258	-1,6496	0,028814099	XR_108881	LOC100506966	uncharacterized LOC100506966	
7,47	7,3	7,28	6,62	6,77	6,49	163,4323	99,127	-1,6487	0,00195809	NM_003270	TSPAN6	tetraspanin 6	
7,48	7,51	7,36	6,7	6,76	6,73	175,028	106,168	-1,6486	0,000256337	ENST00000331373	SGK196	protein kinase-like protein SgK196	
7,75	7,93	7,66	6,95	7,12	7,12	220,465	133,948	-1,6459	0,00290603	NM_015916	CALHM2	calcium homeostasis modulator 2	
6,42	6,71	6,76	6,13	5,9	5,7	99,567	60,5777	-1,6436	0,011319789	NM_018295	TMEM140	transmembrane protein 140	
8,6	8,54	8,39	7,89	7,78	7,72	365,2337	222,613	-1,6407	0,001203881	NM_031476	CRISPLD2	cysteine-rich secretory protein LCCL domain containing 2	
6,7	6,31	6,57	5,88	5,72	5,86	92,773	56,561	-1,6402	0,008288815	NM_001198670	TMEM136	transmembrane protein 136	Yes

Pre-miR-Ctrl (Log2)			Pre-miR-132 (Log2)			Average		Fold Change (miR-132 vs Ctrl)	p-value	Gene Assignment			Predicted miR-132 binding sites
Sample 1	Sample 2	Sample 3	Sample 1	Sample 2	Sample 3	Pre-miR-Ctrl	Pre-miR-132			Public id	Symbol	Name	
8,29	8,22	8,46	7,74	7,46	7,64	321,1023	196,4393	-1,6346	0,003068937	ENST00000522510	OSR2	odd-skipped related 2 (Drosophila)	
5,26	4,62	5,19	4,44	4,29	4,3	33,1377	20,322	-1,6306	0,042451466	ENST00000560415	LOC10050557	uncharacterized LOC100505573	
10,33	10,38	10,41	9,72	9,63	9,66	1326,7767	814,904	-1,6281	4,00436E-05	NM_016441	CRIM1	cysteine rich transmembrane BMP regulator 1 (chordin-like)	
5,68	5,95	6,03	5,29	5,32	4,95	59,4777	36,6603	-1,6224	0,011185782	ENST00000379705	TRPC4	transient receptor potential cation channel, subfamily C, member	
7,73	7,63	7,8	7,06	7,04	6,97	211,085	130,1337	-1,6221	0,000435799	NM_000321	RB1	retinoblastoma 1	Yes
7,89	8,14	8,09	7,23	7,41	7,4	263,9247	163,0307	-1,6189	0,002606501	ENST00000374316	ITPR3	inositol 1,4,5-trisphosphate receptor, type 3	
5,51	5,1	5,48	4,79	4,76	4,47	41,4997	25,641	-1,6185	0,016777789	NM_004636	SEMA3B	sema domain, immunoglobulin domain (Ig), short basic domain, s	
6,38	6,32	6,06	5,48	5,52	5,69	76,6323	47,3813	-1,6174	0,005982403	ENST00000272233	RHOB	ras homolog family member B	
5,03	5,51	5,38	4,25	5	4,53	39,9617	24,71	-1,6172	0,047804044	NM_015379	BRI3	brain protein I3	Yes
7,38	7,18	7,28	6,62	6,58	6,57	155,666	96,3467	-1,6157	0,000713392	ENST00000489418	SLBP	stem-loop binding protein	Yes
5,89	5,5	5,67	4,77	5,19	5,02	51,8237	32,0783	-1,6155	0,015474237	NM_014272	ADAMTS7	ADAM metallopeptidase with thrombospondin type 1 motif, 7	
6,07	5,76	5,8	5,15	5,27	5,16	59,0297	36,615	-1,6122	0,006025569	NM_005516	HLA-E	major histocompatibility complex, class I, E	
8,87	9,09	9,02	8,38	8,22	8,32	510,6623	316,963	-1,6111	0,001457497	ENST00000309446	KLF7	Kruppel-like factor 7 (ubiquitous)	Yes
9,15	9,15	9,19	8,48	8,47	8,48	573,4237	356,232	-1,6097	2,26104E-06	NM_020428	SLC44A2	solute carrier family 44, member 2	
7,89	7,81	7,88	7,1	7,18	7,25	232,3953	144,805	-1,6049	0,000121174	ENST00000535512	PLSCR3	phospholipid scramblase 3	
7,84	7,4	7,67	7,04	7,01	6,84	200,56	125,0173	-1,6043	0,014352801	NM_005338	HIP1	huntingtin interacting protein 1	
5,48	5,83	5,71	4,94	4,93	5,12	51,288	31,9853	-1,6035	0,007348885	NM_001166034	SBSN	suprabasin	
9,19	9,28	9,31	8,35	8,68	8,69	613,4897	383,1457	-1,6012	0,002030317	NM_001199570	CLIP3	CAP-GLY domain containing linker protein 3	
8,52	8,46	8,47	7,61	7,89	7,9	357,94	223,808	-1,5993	0,00085748	NM_032271	TRAF7	TNF receptor-associated factor 7, E3 ubiquitin protein ligase	
6,94	6,73	6,92	6,11	6,32	6,13	116,678	72,9997	-1,5983	0,00229383	NM_014737	RASSF2	Ras association (RalGDS/AF-6) domain family member 2	
8,48	8,58	8,6	7,94	7,82	7,88	375,9193	235,704	-1,5949	0,000226696	NM_015138	RTF1	Rtf1, Paf1/RNA polymerase II complex component, homolog (S. c	
7,61	7,73	7,74	6,69	7,04	7,28	207,15	130,0887	-1,5924	0,008916186	NM_000565	IL6R	interleukin 6 receptor	
5,83	5,8	5,79	5,37	4,93	5,08	55,977	35,2213	-1,5893	0,00308094	NM_001483	GBAS	glioblastoma amplified sequence	Yes
7,71	7,44	7,6	6,9	6,96	6,9	192,3467	121,1187	-1,5881	0,002454263	ENST00000519389	HEPH	hephaestin	
7,25	7,28	7,18	6,45	6,55	6,7	150,8817	95,032	-1,5877	0,000616392	NM_001256658	TEAD2	TEA domain family member 2	
6,65	6,57	6,68	5,82	6,1	5,97	99,3247	62,59	-1,5869	0,000900182	NM_198236	ARHGEF11	Rho guanine nucleotide exchange factor (GEF) 11	Yes
7,51	7,62	7,84	6,92	6,92	7,14	202,708	127,7447	-1,5868	0,008167056	NM_021962	ABR	active BCR-related	
6,59	6,67	6,29	5,76	5,97	5,85	92,138	58,185	-1,5835	0,010784461	NM_006989	RASA4	RAS p21 protein activator 4	
10,09	10,22	10,18	9,45	9,57	9,49	1147,5583	726,1883	-1,5802	0,000278904	NM_007038	ADAMTS5	ADAM metallopeptidase with thrombospondin type 1 motif, 5	
7,81	7,96	7,98	7,31	7,17	7,3	241,9623	153,4253	-1,5771	0,000903757	NM_017583	TRIM44	tripartite motif containing 44	Yes
6,72	6,9	6,71	6,21	6,21	5,93	109,8463	69,675	-1,5766	0,003436392	NM_005388	PDCL	phosducin-like	
6,35	6,29	6,29	5,74	5,5	5,71	79,3567	50,349	-1,5761	0,000487003	NM_014722	FAM65B	family with sequence similarity 65, member B	
6,81	6,9	6,69	6,31	5,98	6,13	111,628	70,8317	-1,576	0,003542721	NM_022765	MICAL1	microtubule associated monoxygenase, calponin and LIM domain	
7,39	7,19	7,07	6,59	6,75	6,33	149,3707	94,8067	-1,5755	0,012212617	NM_020214	PARP6	poly (ADP-ribose) polymerase family, member 6	
6,72	6,25	6,72	5,84	5,82	6,1	95,6497	60,7897	-1,5735	0,029580372	NM_022827	SPATA20	spermatogenesis associated 20	
6,1	6,52	6,39	5,57	5,84	5,66	81,4107	51,7833	-1,5721	0,016010414	ENST00000262366	GLIS2	GLIS family zinc finger 2	
7,79	7,78	7,94	7,15	7,02	7,37	228,8957	145,7443	-1,5705	0,003410986	NM_001130145	YAP1	Yes-associated protein 1	
5,12	5,16	5,2	4,46	4,32	4,72	35,7623	22,779	-1,57	0,002730671	NR_026052	MGC2752	CENPB DNA-binding domains containing 1 pseudogene	
6,01	5,76	5,98	5,26	5,17	5,38	60,5853	38,6547	-1,5673	0,003716408	NM_018367	ACER3	alkaline ceramidase 3	
5,56	5,44	5,34	4,85	4,5	5,01	43,6973	27,8967	-1,5664	0,009779436	NM_001080509	TSPAN11	tetraspanin 11	

Pre-miR-Ctrl (Log2)			Pre-miR-132 (Log2)			Average		Fold Change (miR-132 vs Ctrl)	p-value	Gene Assignment			Predicted miR-132 binding sites
Sample 1	Sample 2	Sample 3	Sample 1	Sample 2	Sample 3	Pre-miR-Ctrl	Pre-miR-132			Public id	Symbol	Name	
9,44	9,38	9,37	8,69	8,85	8,71	674,1843	431,069	-1,564	0,000190207	ENST00000356698	RSPO3	R-spondin 3	
6,4	6,69	6,75	5,78	6,04	6,09	98,4447	62,9557	-1,5637	0,012310873	ENST00000374332	MAN1C1	mannosidase, alpha, class 1C, member 1	
5,81	5,65	5,9	5,12	5,25	5,06	55,3433	35,3967	-1,5635	0,002995381	NM_004938	DAPK1	death-associated protein kinase 1	
7,14	7,57	7,55	6,87	6,57	6,91	172,822	110,7463	-1,5605	0,025056565	NM_030641	APOL6	apolipoprotein L, 6	
9,86	9,96	9,91	9,23	9,27	9,31	962,4567	617,5317	-1,5586	9,06343E-05	ENST00000322088	PPP2R1A	protein phosphatase 2, regulatory subunit A, alpha	
7,14	7,19	7,17	6,31	6,48	6,76	143,6897	92,3293	-1,5563	0,004024169	NM_004756	NUMBL	numb homolog (Drosophila)-like	
6,8	6,57	6,76	6,12	6	6,11	104,941	67,5407	-1,5537	0,002197388	NR_033426	ACAD9	acyl-CoA dehydrogenase family, member 9	Yes
8,23	8,07	8,3	7,72	7,46	7,51	294,7153	189,7287	-1,5534	0,003787007	NM_022977	ACSL4	acyl-CoA synthetase long-chain family member 4	
5,37	5,96	5,53	4,99	4,97	5,06	49,937	32,1597	-1,5528	0,048688345	NM_002198	IRF1	interferon regulatory factor 1	
5,24	5,45	5,17	4,37	4,58	4,96	39,169	25,2403	-1,5518	0,022737151	NM_016084	RASD1	RAS, dexamethasone-induced 1	
6,05	6,36	6,15	5,5	5,51	5,66	73,136	47,1293	-1,5518	0,00655832	NM_002254	KIF3C	kinesin family member 3C	
6,72	6,4	6,58	5,93	5,95	5,94	95,1797	61,394	-1,5503	0,005084371	NM_001083112	GPD2	glycerol-3-phosphate dehydrogenase 2 (mitochondrial)	
6,85	6,88	6,53	6,01	6,05	6,31	108,5183	70,0143	-1,5499	0,014621057	NM_018110	DOK4	docking protein 4	
7,2	7,43	7,15	6,48	6,51	6,88	153,8347	99,3957	-1,5477	0,014460684	ENST00000396593	CARHSP1	calcium regulated heat stable protein 1, 24kDa	
5,72	5,58	5,71	5,03	5,12	4,97	50,9637	32,9297	-1,5477	0,000632805	NR_024071	PLCD1	phospholipase C, delta 1	
6,04	6,26	6,3	5,58	5,66	5,48	73,7437	47,6767	-1,5467	0,003969277	NM_002942	ROBO2	roundabout, axon guidance receptor, homolog 2 (Drosophila)	
8,37	8,5	8,49	7,85	7,79	7,84	350,8063	227,056	-1,545	0,000287964	NM_001008211	OPTN	optineurin	
7,25	7,35	7,38	6,83	6,6	6,67	160,645	104,2023	-1,5417	0,001026463	NM_012320	PLA2G15	phospholipase A2, group XV	
5,4	5,16	5,73	4,6	4,92	4,93	43,6843	28,3363	-1,5416	0,048048189	XR_111050	LOC100509635	uncharacterized LOC100509635	
9,35	9,13	9,12	8,61	8,59	8,54	589,7537	382,7607	-1,5408	0,002911043	NM_018222	PARVA	parvin, alpha	
5,87	5,75	5,6	5,18	4,89	5,27	53,6017	34,8297	-1,539	0,008826058	ENST00000450736	SLC16A5	solute carrier family 16, member 5 (monocarboxylic acid transport	
5,4	5,31	5,04	4,77	4,67	4,45	38,265	24,866	-1,5388	0,01393063	NM_000387	SLC25A20	solute carrier family 25 (carnitine/acylcarnitine translocase), memb	Yes
6,42	6,33	6,3	5,88	5,81	5,47	81,623	53,1063	-1,537	0,004392499	NM_001083	PDE5A	phosphodiesterase 5A, cGMP-specific	
8,26	8,02	8,09	7,24	7,61	7,64	279,536	181,998	-1,5359	0,009487901	XR_111692	LOC100287562	uncharacterized LOC100287562	
8,15	8,26	8,22	7,59	7,6	7,59	296,259	193,1187	-1,5341	9,68563E-05	NM_016513	ICK	intestinal cell (MAK-like) kinase	
5,94	6,01	6,18	5,29	5,37	5,61	66,1143	43,1063	-1,5337	0,006541503	ENST00000355090	ABCA2	ATP-binding cassette, sub-family A (ABC1), member 2	
7,79	7,61	7,59	7,24	6,97	6,92	203,1183	132,5427	-1,5325	0,005744356	NM_001142307	GTF2H1	general transcription factor IIH, polypeptide 1, 62kDa	Yes
5,33	5,08	5,19	4,49	4,66	4,61	36,851	24,0573	-1,5318	0,0032581	NM_130783	TSPAN18	tetraspanin 18	
6,14	6,19	6,23	5,67	5,59	5,45	72,864	47,5983	-1,5308	0,000518662	ENST00000523483	SDCBP	syndecan binding protein (syntenin)	Yes
9,89	9,87	9,82	9,37	9,17	9,19	929,4923	607,262	-1,5306	0,000448194	NM_001128619	LUZP6	leucine zipper protein 6	
5,63	5,16	5,57	5,11	4,77	4,64	44,26	28,9173	-1,5306	0,041360179	ENST00000370828	GPC4	glypican 4	
6,43	6,4	6,26	5,72	5,54	5,98	82,437	54,1187	-1,5233	0,007501435	NM_001077525	MTMR14	myotubularin related protein 14	
6,51	6,45	6,6	6,04	5,87	5,83	91,8573	60,39	-1,5211	0,001296534	NM_001031804	MAF	v-maf musculoaponeurotic fibrosarcoma oncogene homolog (avia	
5,47	5,51	5,38	4,74	4,99	4,81	43,8457	28,851	-1,5197	0,001410735	NM_001031738	TMEM150A	transmembrane protein 150A	
11,22	11,27	11,31	10,71	10,65	10,63	2464,5963	1622,1993	-1,5193	8,53278E-05	NM_001199671	CALU	calumenin	Yes
7,58	7,48	7,37	6,84	6,92	6,87	178,4297	117,5427	-1,518	0,001398784	NM_001005388	NFASC	neurofascin	
6,34	6,41	6,43	5,86	5,86	5,65	84,089	55,4583	-1,5163	0,000727475	NM_003821	RIPK2	receptor-interacting serine-threonine kinase 2	
5,7	6,23	5,91	5,27	5,55	5,25	62,3913	41,164	-1,5157	0,044343676	NM_001166347	SLC26A11	solute carrier family 26, member 11	
5,94	6,02	5,98	5,43	5,38	5,33	63,135	41,6593	-1,5155	8,09966E-05	NM_001122679	ODZ2	odz, odd Oz/ten-m homolog 2 (Drosophila)	
6,63	6,41	6,64	5,99	6,01	5,89	94,6043	62,435	-1,5152	0,003101883	NM_001251978	RCAN3	RCAN family member 3	

Pre-miR-Ctrl (Log2)			Pre-miR-132 (Log2)			Average		Fold Change (miR-132 vs Ctrl)		Gene Assignment			Predicted miR-132 binding sites
Sample 1	Sample 2	Sample 3	Sample 1	Sample 2	Sample 3	Pre-miR-Ctrl	Pre-miR-132		p-value	Public id	Symbol	Name	
5,34	5,01	5,29	4,72	4,46	4,67	37,2837	24,607	-1,5152	0,011728296	NM_001010897	SERP2	stress-associated endoplasmic reticulum protein family member 2	
8,94	9,29	9,26	8,26	8,79	8,62	576,748	380,8793	-1,5143	0,02870053	ENST00000372868	WISP2	WNT1 inducible signaling pathway protein 2	
6,81	6,87	6,59	6,17	6,14	6,18	108,504	71,677	-1,5138	0,004187119	ENST00000458276	ABHD5	abhydrolase domain containing 5	
4,97	5,36	5,21	4,37	4,65	4,73	36,475	24,1077	-1,513	0,020531859	NM_001143981	CHRDL1	chordin-like 1	
6,93	6,8	6,94	6,19	6,44	6,24	118,718	78,472	-1,5129	0,001981762	NM_001142550	WDR47	WD repeat domain 47	
5,39	5,45	5,45	4,82	4,87	4,81	43,1197	28,5133	-1,5123	3,09593E-05	NR_003109	TRNAU1AP	tRNA selenocysteine 1 associated protein 1	Yes
6,09	6,25	6,32	5,51	5,65	5,72	74,7073	49,4977	-1,5093	0,003397683	NM_001100913	PACS2	phosphofurin acidic cluster sorting protein 2	
7,5	7,64	7,63	6,98	6,9	7,11	192,8577	127,9357	-1,5075	0,001295037	NM_013449	BAZ2A	bromodomain adjacent to zinc finger domain, 2A	
6,75	6,73	7,01	6,25	6,21	6,27	114,226	75,7697	-1,5075	0,006537649	NM_005461	MAFB	v-maf musculoaponeurotic fibrosarcoma oncogene homolog B (av	
7,5	7,38	7,65	6,81	7,04	6,91	182,815	121,3547	-1,5065	0,005759168	NM_007039	PTPN21	protein tyrosine phosphatase, non-receptor type 21	
7,34	7,59	7,51	6,8	6,96	6,92	178,989	119,0083	-1,504	0,003629957	NM_213560	PKN1	protein kinase N1	
6,46	6,27	6,66	5,87	5,82	5,96	88,7773	59,076	-1,5028	0,014062644	NR_027297	HOMER3	homer homolog 3 (Drosophila)	
6,31	6,61	6,68	5,97	6,01	5,88	93,1863	62,0067	-1,5028	0,012643223	NM_017631	DDX60	DEAD (Asp-Glu-Ala-Asp) box polypeptide 60	
5,9	6,26	6,09	5,47	5,43	5,61	68,1577	45,425	-1,5004	0,011854274	ENST00000216039	JOSD1	Josephin domain containing 1	Yes
6,78	6,72	6,66	6,03	6,14	6,23	105,4803	70,3093	-1,5002	0,00074257	NM_014732	KIAA0513	KIAA0513	
4,38	4,74	4,75	5,39	5,28	4,95	24,8177	37,2323	1,5002	0,031996551	NM_020784	TXNDC16	thioredoxin domain containing 16	
5,18	4,92	5,17	5,75	5,66	5,63	34,176	51,3007	1,5011	0,001860797	NM_182503	ADAT2	adenosine deaminase, tRNA-specific 2	
5,02	4,68	5,11	5,48	5,36	5,74	30,872	46,3827	1,5024	0,027156227	AF422192	LOC10028816(	esophagus cancer-related gene-2 interaction susceptibility protein	
9,39	9,59	9,41	10,01	10,05	10,1	707,298	1062,9097	1,5028	0,000666195	NM_013417	IARS	isoleucyl-tRNA synthetase	
5,66	5,64	5,61	6,19	6,4	6,07	49,7567	74,88	1,5049	0,007873937	NR_003610	PDXDC2P	pyridoxal-dependent decarboxylase domain containing 2, pseudog	
7	6,92	6,75	7,39	7,49	7,57	118,91	179,1727	1,5068	0,002368528	NR_046315	BLOC1S2	biogenesis of lysosomal organelles complex-1, subunit 2	
4,65	4,59	4,71	5,2	5,45	5,05	25,1213	37,8663	1,5073	0,01574947	NM_014496	RPS6KA6	ribosomal protein S6 kinase, 90kDa, polypeptide 6	
7,19	7,21	6,63	7,73	7,51	7,63	131,0393	197,5573	1,5076	0,02170431	NM_001827	CKS2	CDC28 protein kinase regulatory subunit 2	
9,68	9,78	9,82	10,31	10,25	10,49	867,785	1308,4537	1,5078	0,003430789	ENST00000524558	SERPINH1	serpin peptidase inhibitor, clade H (heat shock protein 47), memb	
5,08	4,61	5,24	5,52	5,59	5,67	32,0123	48,323	1,5095	0,018111908	ENST00000457714	NLGN1	neuroligin 1	
5,81	6,39	6,04	6,76	6,63	6,69	68,589	103,559	1,5098	0,015099105	ENST00000498004	CA5B	carbonic anhydrase VB, mitochondrial	
5,9	5,6	5,34	6,24	6,05	6,37	49,5737	74,8507	1,5099	0,026095897	ENST00000369158	HIST2H3D	histone cluster 2, H3d	
4,58	4,62	4,24	5,13	5,11	5,01	22,468	33,925	1,5099	0,004537176	AK127211	DNAH10OS	dynein, axonemal, heavy chain 10 opposite strand	
4,8	4,46	4,91	5,24	5,26	5,48	26,644	40,2477	1,5106	0,013949134	NM_005522	HOXA1	homeobox A1	
5,99	6,63	6,38	6,77	6,97	7,1	81,9627	123,8967	1,5116	0,032830043	ENST00000261326	MOCOS	molybdenum cofactor sulfurase	
9,15	9,35	9,45	9,71	9,93	10,09	640,0293	967,6493	1,5119	0,016496735	NM_012098	ANGPTL2	angiopoietin-like 2	
5,93	5,78	5,45	6,24	6,37	6,38	53,21	80,527	1,5134	0,008330904	NM_006461	SPAG5	sperm associated antigen 5	
5,38	5,16	4,98	5,85	5,75	5,74	36,3183	54,981	1,5139	0,004420089	ENST00000433078	C7orf23	chromosome 7 open reading frame 23	
5,45	4,83	5,24	5,65	5,8	5,92	36,6493	55,492	1,5141	0,024471759	ENST00000394715	ZBTB25	zinc finger and BTB domain containing 25	
6,61	6,36	6,8	7,22	7,22	7,16	97,0833	147,0617	1,5148	0,0045502	AF400488	SNRPN	small nuclear ribonucleoprotein polypeptide N	
5,9	5,55	5,87	6,44	6,29	6,41	55,0167	83,3693	1,5153	0,004301109	NM_015271	TRIM2	tripartite motif containing 2	
7,7	7,42	7,55	8,33	7,9	8,22	188,865	286,2743	1,5158	0,022219358	NR_027337	HIST2H2BA	histone cluster 2, H2ba (pseudogene)	
6,17	6,26	6,17	6,74	6,79	6,87	73,549	111,5073	1,5161	0,000334466	ENST00000550765	FKBP11	FK506 binding protein 11, 19 kDa	
4,94	4,83	4,75	5,28	5,4	5,63	28,6827	43,5333	1,5178	0,0112204	NM_001025366	VEGFA	vascular endothelial growth factor A	
4,98	4,85	5,04	5,62	5,57	5,49	31,0997	47,209	1,518	0,000717547	NR_003237	SNORD113-9	small nucleolar RNA, C/D box 113-9	

Pre-miR-Ctrl (Log2)			Pre-miR-132 (Log2)			Average		Fold Change (miR-132 vs Ctrl)	p-value	Gene Assignment			Predicted miR-132 binding sites
Sample 1	Sample 2	Sample 3	Sample 1	Sample 2	Sample 3	Pre-miR-Ctrl	Pre-miR-132			Public id	Symbol	Name	
5,32	5,41	5,45	6,15	5,93	5,9	42,0593	63,8983	1,5192	0,004304102	NM_003503	CDC7	cell division cycle 7 homolog (S. cerevisiae)	
7,97	8,01	7,88	8,64	8,49	8,54	248,027	376,8957	1,5196	0,000640887	NM_016657	KDELR3	KDEL (Lys-Asp-Glu-Leu) endoplasmic reticulum protein retention	
7,17	7,04	7,08	7,76	7,65	7,7	136,968	208,5193	1,5224	0,000263818	NM_005080	XBP1	X-box binding protein 1	
5,08	5,13	4,92	5,83	5,59	5,53	33,0387	50,42	1,5261	0,008292542	ENST00000268138	C15orf42	chromosome 15 open reading frame 42	
4,34	4,13	4,61	5,04	4,96	4,95	20,727	31,645	1,5268	0,006584287	ENST00000493990	ARL6	ADP-ribosylation factor-like 6	
6,15	6,27	6,27	6,74	6,7	7,06	75,1187	114,765	1,5278	0,014474738	NM_182513	SPC24	SPC24, NDC80 kinetochore complex component, homolog (S. ce	
8,2	8,26	8,1	8,74	8,88	8,78	291,6653	446,0957	1,5295	0,000647159	NM_001243246	LEPRE1	leucine proline-enriched proteoglycan (leprecan) 1	
5,16	5,19	5,35	5,95	5,92	5,66	37,681	57,6437	1,5298	0,006825162	NM_153704	TMEM67	transmembrane protein 67	
6,02	5,88	5,73	6,39	6,55	6,54	58,9537	90,2067	1,5301	0,002574952	NM_015846	MBD1	methyl-CpG binding domain protein 1	
5,42	5,31	5,16	5,93	6,11	5,67	39,4127	60,318	1,5304	0,020652815	NM_006670	TPBG	trophoblast glycoprotein	
4,5	4,8	4,23	5,31	4,91	5,18	23,0833	35,3293	1,5305	0,033586621	NM_020315	PDXP	pyridoxal (pyridoxine, vitamin B6) phosphatase	
5,37	4,94	5,26	5,9	5,72	5,82	36,79	56,3057	1,5305	0,006569036	NM_001102559	PPAPDC1B	phosphatidic acid phosphatase type 2 domain containing 1B	
4,82	4,64	4,43	5,5	5,01	5,21	24,9117	38,164	1,532	0,036036512	NM_001185181	PFDN6	prefoldin subunit 6	
6,09	5,63	5,96	6,61	6,72	6,18	59,964	91,8687	1,5321	0,048288229	NM_020652	ZNF286A	zinc finger protein 286A	
8,17	7,93	7,86	8,34	8,79	8,66	254,7383	390,3927	1,5325	0,025102328	NR_037447	MIR3676	microRNA 3676	
8,14	8,02	7,95	8,7	8,53	8,73	262,9807	403,377	1,5339	0,002115212	NM_021982	SEC24A	SEC24 family, member A (S. cerevisiae)	
8,29	8,43	8,7	9,04	9,1	9,16	357,9203	549,0643	1,534	0,004483914	ENST00000295213	SPATA18	spermatogenesis associated 18	
7,44	7,41	7,39	7,94	8,1	8,05	170,4823	261,658	1,5348	0,000459464	BC019843	C12orf49	chromosome 12 open reading frame 49	
6,75	6,69	7,04	7,43	7,49	7,45	114,1613	175,6893	1,539	0,002465165	ENST00000370986	GADD45A	growth arrest and DNA-damage-inducible, alpha	
6,28	5,99	6,03	6,66	6,62	6,89	68,8703	106,0293	1,5396	0,008654073	NM_014573	TMEM97	transmembrane protein 97	
9,38	9,48	9,4	9,97	10,03	10,13	685,328	1056,333	1,5414	0,000578066	NM_001605	AARS	alanyl-tRNA synthetase	
5	4,9	4,79	5,31	5,81	5,4	29,8407	45,9993	1,5415	0,037145568	AK303556	LOC100505620	uncharacterized LOC100505620	
5,05	5,21	5,1	5,84	5,87	5,5	34,813	53,674	1,5418	0,012562998	NM_001145293	NGLY1	N-glycanase 1	
7,32	7,34	7,37	8,13	7,98	7,78	162,408	250,8027	1,5443	0,007250675	NM_006948	HSPA13	heat shock protein 70kDa family, member 13	
5,06	5,36	5,32	5,74	6,14	5,72	38,1253	58,8927	1,5447	0,029976163	ENST00000281830	KCNE4	potassium voltage-gated channel, Isk-related family, member 4	
6,46	6,48	6,36	7,2	6,94	7,04	86,4793	133,806	1,5473	0,003098695	NM_018136	ASPM	asp (abnormal spindle) homolog, microcephaly associated (Droso	
6,87	6,71	6,79	7,2	7,46	7,58	110,774	171,481	1,548	0,010765012	NM_206538	EMC10	ER membrane protein complex subunit 10	
6,32	6,54	6,52	7,09	7,17	7,02	88,24	136,6777	1,5489	0,001176318	NR_026641	INTS2	integrator complex subunit 2	
5,13	5,16	5,22	5,9	5,57	5,91	36,0137	55,7827	1,5489	0,009210287	NR_040001	LOC375295	uncharacterized LOC375295	
5,38	5	5,17	5,81	5,82	5,84	36,5483	56,626	1,5493	0,002052831	NM_006845	KIF2C	kinesin family member 2C	
4,73	4,22	4,67	5,27	4,99	5,29	23,5437	36,4963	1,5502	0,019339464	NM_014254	TMEM5	transmembrane protein 5	
8,66	8,55	8,68	9,32	9,19	9,28	396,485	614,9613	1,551	0,000368643	ENST00000504154	SLIT2	slit homolog 2 (Drosophila)	
5,73	5,64	5,87	6,3	6,53	6,31	53,8093	83,515	1,5521	0,004364745	ENST00000403410	TMEM45A	transmembrane protein 45A	
4,2	4,37	4,61	5,05	5,04	5,03	21,159	32,9	1,5549	0,002653618	AK002146	LOC153684	uncharacterized LOC153684	
7,28	7,27	7,5	7,9	8,01	8,06	163,593	254,5027	1,5557	0,001629995	ENST00000285021	XPC	xeroderma pigmentosum, complementation group C	
5,17	5,09	5,48	6,13	5,72	5,8	38,2313	59,4867	1,556	0,027325037	NM_003509	HIST1H2AI	histone cluster 1, H2ai	
6,46	6,63	6,39	7,29	6,92	7,17	90,3147	140,5333	1,556	0,011339436	NM_001201362	HMGN3	high mobility group nucleosomal binding domain 3	
5,21	5,68	5,81	6,42	6,19	6,05	48,1283	74,9643	1,5576	0,029180283	NM_012189	CABYR	calcium binding tyrosine-(Y)-phosphorylation regulated	
4,6	4,55	4,79	5,26	5,42	5,18	25,1137	39,1283	1,558	0,00385701	NM_004412	TRDMT1	tRNA aspartic acid methyltransferase 1	
7,23	6,75	7,18	7,81	7,67	7,64	134,2557	209,178	1,5581	0,008350559	NM_001134445	DDAH1	dimethylarginine dimethylaminohydrolase 1	

Pre-miR-Ctrl (Log2)			Pre-miR-132 (Log2)			Average		Fold Change (miR-132 vs Ctrl)	p-value	Gene Assignment			Predicted miR-132 binding sites
Sample 1	Sample 2	Sample 3	Sample 1	Sample 2	Sample 3	Pre-miR-Ctrl	Pre-miR-132			Public id	Symbol	Name	
8,97	9,3	9,16	9,89	9,5	9,94	567,9533	885,063	1,5583	0,023711164	NR_006881	SNORD3C	small nucleolar RNA, C/D box 3C	
9,35	9,14	9,23	9,95	9,69	9,99	605,747	944,0157	1,5584	0,006441333	NM_001008397	GPX8	glutathione peroxidase 8 (putative)	
6,8	7,07	6,96	7,59	7,65	7,52	123,4313	192,3573	1,5584	0,001155188	NM_006502	POLH	polymerase (DNA directed), eta	
5,28	5,51	5,48	6,26	5,89	6,03	43,0187	67,0953	1,5597	0,011879248	NM_001201480	OSBPL6	oxysterol binding protein-like 6	
6,4	6,05	6,23	7,02	6,72	6,87	75,2557	117,3923	1,5599	0,008658143	ENST00000554684	SMEK1	SMEK homolog 1, suppressor of mek1 (Dictyostelium)	
4,72	4,69	4,96	5,34	5,26	5,68	27,7643	43,3637	1,5618	0,022931756	NM_003256	TIMP4	TIMP metallopeptidase inhibitor 4	
8,4	8,45	8,13	8,98	8,92	9,03	322,5463	504,0303	1,5627	0,001684449	ENST00000265748	ANLN	anillin, actin binding protein	
4,74	4,13	4,45	5,11	5,14	5,07	22,0297	34,4623	1,5644	0,010057372	NR_024452	LOC100134259	uncharacterized LOC100134259	
5,38	5,39	5,3	6,13	5,95	5,92	40,991	64,1343	1,5646	0,001677307	ENST00000423383	CENPI	centromere protein I	
6,87	6,36	6,38	7,26	7,08	7,26	94,1317	147,284	1,5647	0,014610364	NM_016059	PPIL1	peptidylprolyl isomerase (cyclophilin)-like 1	
4,37	4,3	4,73	5,27	4,8	5,26	22,3047	34,921	1,5656	0,037786719	ENST00000356316	CAPN3	calpain 3, (p94)	
6,36	6,3	6,39	7,21	6,88	6,88	81,599	127,8747	1,5671	0,010522152	NR_003232	SNORD113-4	small nucleolar RNA, C/D box 113-4	
8,16	8,31	7,77	8,74	8,64	8,85	273,8887	429,3123	1,5675	0,010643569	NM_005733	KIF20A	kinesin family member 20A	
6,66	6,88	6,86	7,47	7,47	7,42	111,6903	175,281	1,5693	0,000360579	NM_015187	SEL1L3	sel-1 suppressor of lin-12-like 3 (C. elegans)	
7,31	7,27	6,9	7,76	7,78	7,92	144,1513	226,2503	1,5695	0,005150328	ENST00000338825	EIF4EBP1	eukaryotic translation initiation factor 4E binding protein 1	
6,04	6,01	5,84	6,68	6,7	6,46	62,5087	98,18	1,5707	0,003399955	ENST00000301905	PBK	PDZ binding kinase	
5,57	5,41	5,46	5,94	6,11	6,33	44,68	70,3043	1,5735	0,011052606	NM_198596	SULF2	sulfatase 2	
4,89	4,38	4,74	5,54	5,19	5,28	25,7317	40,6283	1,5789	0,020197497	NM_000270	PNP	purine nucleoside phosphorylase	
4,75	4,79	4,53	5,33	5,18	5,53	25,8923	40,894	1,5794	0,009586784	NM_001130914	BTG3	BTG family, member 3	
5,37	5,14	5,21	5,87	5,97	5,87	37,8767	59,8843	1,581	0,000654113	BC080646	SNRPN	small nuclear ribonucleoprotein polypeptide N	
5,81	5,87	5,52	6,57	6,27	6,35	53,4917	84,5847	1,5813	0,009290075	NM_014875	KIF14	kinesin family member 14	
6,73	6,88	6,92	7,47	7,59	7,46	115,0107	182,0117	1,5826	0,000667344	NM_003882	WISP1	WNT1 inducible signaling pathway protein 1	
7,44	7,49	7,4	8,11	8,09	8,12	174,1037	275,655	1,5833	9,0709E-06	NM_013231	FLRT2	fibronectin leucine rich transmembrane protein 2	
9,44	9,45	9,42	10,15	10,07	10,08	693,0043	1097,8317	1,5842	3,35408E-05	NM_006854	KDELR2	KDEL (Lys-Asp-Glu-Leu) endoplasmic reticulum protein retention	
7,77	7,68	7,33	8,27	8,19	8,35	194,749	309,0037	1,5867	0,00461836	ENST00000300093	PLK1	polo-like kinase 1	
7,29	7,16	7,1	7,96	7,76	7,83	145,566	231,1037	1,5876	0,001508345	ENST00000310823	ADAM17	ADAM metallopeptidase domain 17	
7,97	7,78	8,03	8,7	8,53	8,56	243,968	387,644	1,5889	0,001628869	NM_002860	ALDH18A1	aldehyde dehydrogenase 18 family, member A1	
4,78	4,37	4,29	5,27	5,3	4,89	22,5713	35,878	1,5895	0,028804728	NM_001033719	ZNF404	zinc finger protein 404	
6,05	5,87	5,41	6,61	6,51	6,27	55,7533	88,664	1,5903	0,023563191	NR_003209	SNORD114-16	small nucleolar RNA, C/D box 114-16	
9,06	8,87	8,88	9,65	9,59	9,59	490,92	781,5953	1,5921	0,000268722	ENST00000259056	GALNT5	UDP-N-acetyl-alpha-D-galactosamine:polypeptide N-acetylgalacto	
4,2	4,44	4,38	4,97	5,27	4,76	20,302	32,341	1,593	0,026271654	ENST00000395321	ALDOC	aldolase C, fructose-bisphosphate	
5,25	4,92	5,26	5,88	5,78	5,81	35,5493	56,6477	1,5935	0,001864091	NM_032016	STARD3NL	STARD3 N-terminal like	
7,81	7,74	7,65	8,39	8,35	8,48	213,016	339,601	1,5943	0,000371945	NM_018184	ARL8B	ADP-ribosylation factor-like 8B	
5,9	5,8	5,61	6,45	6,34	6,55	54,7563	87,3787	1,5958	0,002542128	NM_005787	ALG3	asparagine-linked glycosylation 3, alpha-1,3- mannosyltransferase	
4,9	5,08	5	5,55	5,9	5,54	31,894	51,0307	1,6	0,0130471	NR_047669	FAM173B	family with sequence similarity 173, member B	
5,75	5,69	5,84	6,62	6,39	6,3	54,2413	87,006	1,6041	0,005767169	ENST00000498286	MTO1	mitochondrial translation optimization 1 homolog (S. cerevisiae)	
6,71	6,61	6,58	7,32	7,48	7,14	99,3473	159,7857	1,6084	0,005634298	NM_017752	TBC1D8B	TBC1 domain family, member 8B (with GRAM domain)	
7,15	7,45	7,27	7,97	7,99	7,99	157,0737	253,0653	1,6111	0,00057253	NM_002079	GOT1	glutamic-oxaloacetic transaminase 1, soluble (aspartate aminotrar	
6,03	6,02	5,71	6,69	6,47	6,68	60,8613	98,1447	1,6126	0,004282326	ENST00000446531	H2AFV	H2A histone family, member V	
4,62	4,32	4,33	5,4	5,11	4,8	21,5583	34,8723	1,6176	0,039433923	NM_020445	ACTR3B	ARP3 actin-related protein 3 homolog B (yeast)	

Pre-miR-Ctrl (Log2)			Pre-miR-132 (Log2)			Average		Fold Change (miR-132 vs Ctrl)	p-value	Gene Assignment			Predicted miR-132 binding sites
Sample 1	Sample 2	Sample 3	Sample 1	Sample 2	Sample 3	Pre-miR-Ctrl	Pre-miR-132			Public id	Symbol	Name	
6,41	5,98	5,87	6,77	6,88	6,75	68,88	111,5187	1,619	0,008293063	NM_018410	HJURP	Holliday junction recognition protein	
4,92	4,82	5,1	5,68	5,44	5,8	30,939	50,1313	1,6203	0,008755715	NM_012118	CCRN4L	CCR4 carbon catabolite repression 4-like (S. cerevisiae)	
6,07	6,05	5,85	6,82	6,49	6,74	63,7063	103,2537	1,6208	0,006334889	NM_145061	SKA3	spindle and kinetochore associated complex subunit 3	
6,49	6,51	6,39	7,3	7,03	7,14	88,296	143,1067	1,6208	0,002543491	ENST00000395471	PSPH	phosphoserine phosphatase	
4,62	4,19	4,11	5,11	4,97	4,98	20,0367	32,4783	1,6209	0,007778137	ENST00000480135	CLDN12	claudin 12	
8,61	8,62	8,69	9,28	9,34	9,39	399,0543	646,8853	1,621	0,000098103	NM_014774	KIAA0494	KIAA0494	
5,78	6,09	5,92	6,4	6,63	6,84	61,2053	99,352	1,6233	0,015898483	NM_005067	SIAH2	siah E3 ubiquitin protein ligase 2	
6,34	6,5	6,07	7,07	7,1	6,86	79,5667	129,2377	1,6243	0,006267442	NM_006763	BTG2	BTG family, member 2	
4,63	4,24	4,52	5,33	5,15	5,02	22,2	36,059	1,6243	0,008260056	NM_002703	PPAT	phosphoribosyl pyrophosphate amidotransferase	
5,43	5,24	5,49	6,08	6,1	6,1	41,9483	68,279	1,6277	0,00026342	NM_001137610	FAM86B2	family with sequence similarity 86, member B2	
4,42	4,38	4,2	4,99	5,07	5,06	20,2023	32,9097	1,629	0,000307316	NM_000112	SLC26A2	solute carrier family 26 (sulfate transporter), member 2	
5,22	5,08	4,87	5,84	5,76	5,7	33,4463	54,486	1,6291	0,001649539	ENST00000286614	MMP16	matrix metallopeptidase 16 (membrane-inserted)	
9,58	9,45	9,46	10,29	10	10,3	723,0177	1178,892	1,6305	0,004759079	AK292062	HIST1H2BK	histone cluster 1, H2bk	
6,81	6,34	6,43	7,33	7,23	7,18	93,1457	152,01	1,632	0,005367902	NM_180989	GPR180	G protein-coupled receptor 180	
6,1	6,15	6,09	6,87	6,77	6,82	69,242	113,031	1,6324	5,59482E-05	NM_005147	DNAJA3	DnaJ (Hsp40) homolog, subfamily A, member 3	
5,31	5,11	4,83	5,93	5,8	5,67	34,2163	55,866	1,6327	0,007641991	ENST00000419585	FANCD2	Fanconi anemia, complementation group D2	
7,23	7,17	7,06	7,69	7,95	7,94	142,522	233,1173	1,6357	0,00308193	NM_058219	EXOSC6	exosome component 6	
6,64	6,56	6,6	7,52	7,16	7,23	97,0307	158,894	1,6376	0,00796951	ENST00000406462	GPSM2	G-protein signaling modulator 2	
7,09	6,57	6,73	7,57	7,58	7,42	112,4673	184,205	1,6379	0,006723214	NM_006915	RP2	retinitis pigmentosa 2 (X-linked recessive)	
4,57	4,44	4,6	5,18	5,49	5,05	23,2363	38,1073	1,64	0,014743402	NM_018349	MCTP2	multiple C2 domains, transmembrane 2	
4,76	4,79	4,85	5,7	5,58	5,23	27,867	45,7833	1,6429	0,014370278	NM_000059	BRCA2	breast cancer 2, early onset	
6,97	6,75	7,03	7,79	7,5	7,61	121,2303	199,234	1,6434	0,004700197	NM_024089	KDELC1	KDEL (Lys-Asp-Glu-Leu) containing 1	
5,33	5,27	5,34	6,04	5,99	6,06	39,7713	65,3583	1,6434	2,12024E-05	NM_024090	ELOVL6	ELOVL fatty acid elongase 6	
6,41	6,31	6,31	7,07	6,95	7,16	81,2393	133,6723	1,6454	0,000895085	NM_145041	TMEM106A	transmembrane protein 106A	
5,12	5,67	5,87	6,22	6,44	6,26	48,0583	79,335	1,6508	0,017071154	NM_000621	HTR2A	5-hydroxytryptamine (serotonin) receptor 2A, G protein-coupled	
4,94	5,32	4,91	5,91	5,8	5,66	33,5693	55,469	1,6524	0,006585857	ENST00000464609	ARHGDIG	Rho GDP dissociation inhibitor (GDI) gamma	
4,63	4,27	4,2	5,14	4,78	5,34	20,811	34,413	1,6536	0,033542537	NR_002318	CATSPER2P1	cation channel, sperm associated 2 pseudogene 1	
4,66	4,6	4,69	5,4	5,33	5,4	25,115	41,5573	1,6547	3,46212E-05	ENST00000356113	LIPC	lipase, hepatic	
5,39	5,57	5,19	6,04	6,23	6,08	41,9807	69,503	1,6556	0,002939619	NM_032315	SLC25A33	solute carrier family 25 (pyrimidine nucleotide carrier), member 33	
4,21	4,69	4,51	5,49	4,89	5,19	22,3683	37,0323	1,6556	0,04032141	NR_027049	ZNF788	zinc finger family member 788	
4,29	4,34	4,47	4,89	4,91	5,43	20,6587	34,2757	1,6591	0,038604139	NM_022117	TSPYL2	TSPY-like 2	
7,65	7,68	7,65	8,3	8,51	8,36	202,2607	336,0957	1,6617	0,0008313	NM_021988	UBE2V1	ubiquitin-conjugating enzyme E2 variant 1	
4,69	4,54	4,63	5,37	5,26	5,43	24,6127	40,9283	1,6629	0,000499966	NM_001037540	SCML1	sex comb on midleg-like 1 (Drosophila)	
7,03	7,07	6,93	7,79	7,8	7,64	128,9973	214,5497	1,6632	0,000524958	NM_147188	FBXO22	F-box protein 22	
4,53	4,25	4,72	5,15	5,42	5,16	22,8283	38,0243	1,6657	0,008970176	ENST00000260229	MMP27	matrix metallopeptidase 27	
6	6,06	5,86	6,88	6,67	6,57	62,933	104,8743	1,6664	0,004356398	NM_016448	DTL	denticleless E3 ubiquitin protein ligase homolog (Drosophila)	
8,51	8,6	8,37	9,22	9,2	9,28	361,1407	602,0487	1,6671	0,000242876	NM_002581	PAPPA	pregnancy-associated plasma protein A, pappalysin 1	
5,12	5,24	5,5	5,9	6,22	5,96	39,2743	65,5023	1,6678	0,009063857	NM_020799	STAMBPL1	STAM binding protein-like 1	
5,09	5,03	5,29	5,99	5,85	5,79	35,2853	58,856	1,668	0,001676309	NM_005133	RCE1	RCE1 homolog, prenyl protein protease (S. cerevisiae)	
5,76	6,04	5,86	6,44	6,77	6,67	59,3573	99,263	1,6723	0,005722579	NM_133436	ASNS	asparagine synthetase (glutamine-hydrolyzing)	

Pre-miR-Ctrl (Log2)			Pre-miR-132 (Log2)			Average		Fold Change (miR-132 vs Ctrl)	p-value	Gene Assignment			Predicted miR-132 binding sites
Sample 1	Sample 2	Sample 3	Sample 1	Sample 2	Sample 3	Pre-miR-Ctrl	Pre-miR-132			Public id	Symbol	Name	
5,06	5,14	5,06	5,8	5,81	5,88	33,993	56,9033	1,674	4,21273E-05	NM_032601	MCEE	methylmalonyl CoA epimerase	
4,73	4,9	5,12	5,78	5,61	5,61	30,3903	50,876	1,6741	0,002857133	NM_052954	CYYR1	cysteine/tyrosine-rich 1	
10,57	10,46	10,47	11,31	11,18	11,24	1449,0197	2425,9133	1,6742	0,000176728	NM_005570	LMAN1	lectin, mannose-binding, 1	
7,1	7,2	6,97	7,98	7,75	7,77	136,5287	228,6733	1,6749	0,002393822	ENST00000342502	IKBIP	IKBKB interacting protein	
8,41	8,47	8,44	9,17	9,18	9,21	347,341	582,7637	1,6778	3,28302E-06	ENST00000433473	PPT1	palmitoyl-protein thioesterase 1	
4,69	4,9	4,43	5,31	5,55	5,43	25,742	43,211	1,6786	0,005284248	ENST00000422053	TRIB3	tribbles homolog 3 (Drosophila)	
5,26	5,13	5,36	6,15	5,99	5,85	38,1353	64,0833	1,6804	0,003597763	ENST00000333129	FIGN	fidgetin	
6,18	6,14	6,05	6,76	7,02	6,83	69,7613	117,314	1,6816	0,00206591	NM_001128431	SLC39A14	solute carrier family 39 (zinc transporter), member 14	
5,21	4,95	4,83	5,66	5,93	5,66	32,1223	54,0317	1,6821	0,007026854	NM_001264573	KIF18B	kinesin family member 18B	
6,27	6,29	6,42	7,15	6,99	7,1	80,3493	135,4427	1,6857	0,00042697	NR_046478	FKBP14	FK506 binding protein 14, 22 kDa	
8,51	8,73	8,58	9,42	9,4	9,28	390,6167	660,7583	1,6916	0,000526692	NM_002392	MDM2	Mdm2, p53 E3 ubiquitin protein ligase homolog (mouse)	
6,54	6,75	6,38	7,4	7,27	7,31	94,6583	160,641	1,6971	0,001347413	NM_014750	DLGAP5	discs, large (Drosophila) homolog-associated protein 5	
5,75	5,66	5,6	6,2	6,54	6,54	50,961	86,5417	1,6982	0,006023066	ENST00000361288	TAF11	TAF11 RNA polymerase II, TATA box binding protein (TBP)-assoc	
5,15	4,58	3,99	5,43	5,38	5,44	25,1043	42,7217	1,7018	0,036934487	NR_001447	MT1L	metallothionein 1L (gene/pseudogene)	
6,82	6,82	6,5	7,6	7,43	7,43	105,494	179,6347	1,7028	0,002034539	ENST00000303383	SHCBP1	SHC SH2-domain binding protein 1	
4,83	4,13	4,35	5,07	5,54	5,04	22,115	37,6727	1,7035	0,047619707	AK130707	LOC10012813	uncharacterized LOC100128130	
5,15	5,13	4,94	5,97	5,88	5,67	33,7397	57,4963	1,7041	0,003306112	NM_031217	KIF18A	kinesin family member 18A	
6,64	6,36	6,66	7,36	7,32	7,31	94,3323	160,916	1,7058	0,000466203	NM_181342	FKBP7	FK506 binding protein 7	
7,19	6,59	6,3	7,53	7,29	7,7	107,049	183,086	1,7103	0,038487725	NR_027054	MIR31HG	MIR31 host gene (non-protein coding)	
6,97	7,38	7,3	7,6	8,17	8,17	149,8413	256,6807	1,713	0,033967394	NM_199054	MKNK2	MAP kinase interacting serine/threonine kinase 2	
9,37	9,3	8,82	9,98	9,68	10,19	581,325	999,4467	1,7193	0,025188572	ENST00000364102	RNU5A-8P	RNA, U5A small nuclear 8, pseudogene	
5,37	5,35	5,65	6,41	6,22	6,09	44,118	75,8997	1,7204	0,005394338	ENST00000289352	HIST2H4B	histone cluster 2, H4b	Yes
6,15	6,07	6,36	6,93	6,9	7,11	73,4443	126,5023	1,7224	0,001986276	NM_016262	TUBE1	tubulin, epsilon 1	
5,68	5,68	5,52	6,21	6,5	6,52	49,4743	85,437	1,7269	0,003875829	NM_007175	ERLIN2	ER lipid raft associated 2	
5,8	5,46	5,75	6,28	6,62	6,48	51,183	88,444	1,728	0,005960207	NM_004793	LONP1	lon peptidase 1, mitochondrial	
6,83	6,95	6,84	7,88	7,68	7,39	117,325	202,7907	1,7285	0,012633639	NR_003210	SNORD114-17	small nucleolar RNA, C/D box 114-17	
4,6	4,99	4,93	5,78	5,38	5,74	28,838	50,0123	1,7343	0,011639904	NM_002758	MAP2K6	mitogen-activated protein kinase kinase 6	
4,85	4,81	4,58	5,34	5,86	5,38	26,9363	46,7427	1,7353	0,028088009	NM_001171690	GCAT	glycine C-acetyltransferase	
5,17	4,62	4,93	5,9	5,65	5,59	30,3587	52,6983	1,7359	0,009975691	NM_001123375	HIST2H3D	histone cluster 2, H3d	
5,63	5,19	4,98	6,11	6,22	5,92	39,195	68,054	1,7363	0,012758923	NM_014061	MAGEH1	melanoma antigen family H, 1	
8,67	8,58	8,51	9,49	9,3	9,37	384,851	670,369	1,7419	0,000579834	NM_015696	GPX7	glutathione peroxidase 7	
4,75	4,69	4,5	5,75	5,4	5,14	25,1163	43,7673	1,7426	0,028485512	ENST00000262067	TSPAN13	tetraspanin 13	
7,21	6,81	6,83	7,95	7,69	7,63	124,678	217,2893	1,7428	0,008459035	NM_002426	MMP12	matrix metallopeptidase 12 (macrophage elastase)	
6,53	6,62	6,45	7,39	7,19	7,42	92,7327	161,6677	1,7434	0,001256852	NM_020375	C12orf5	chromosome 12 open reading frame 5	
6,77	6,97	6,87	7,57	7,7	7,75	117,1577	204,4083	1,7447	0,000591999	NM_006623	PHGDH	phosphoglycerate dehydrogenase	
5,74	5,24	5,92	6,56	6,35	6,48	50,5953	88,3963	1,7471	0,007918768	NM_052885	SLC2A13	solute carrier family 2 (facilitated glucose transporter), member 13	
7,79	7,82	7,87	8,62	8,55	8,73	227,0783	397,6193	1,751	0,000340296	NM_002592	PCNA	proliferating cell nuclear antigen	
6,16	6,31	6,56	7,24	7,14	7,11	81,7333	143,4507	1,7551	0,001366556	ENST00000518109	ENPP2	ectonucleotide pyrophosphatase/phosphodiesterase 2	
8,78	8,89	8,91	9,66	9,7	9,66	465,0117	816,5833	1,756	1,92541E-05	NM_139276	STAT3	signal transducer and activator of transcription 3 (acute-phase res	
5,74	5,81	5,64	6,63	6,48	6,52	53,1387	93,3603	1,7569	0,000307535	XR_110188	LOC100507624	uncharacterized LOC100507624	

Pre-miR-Ctrl (Log2)			Pre-miR-132 (Log2)			Average		Fold Change (miR-132 vs Ctrl)	p-value	Gene Assignment			Predicted miR-132 binding sites
Sample 1	Sample 2	Sample 3	Sample 1	Sample 2	Sample 3	Pre-miR-Ctrl	Pre-miR-132			Public id	Symbol	Name	
6,81	6,72	6,92	7,62	7,69	7,59	112,907	198,6307	1,7592	0,000150638	NM_001104587	SLFN11	schlafen family member 11	
6,45	6,1	6,22	7,18	7,02	7,04	76,8547	135,465	1,7626	0,001336298	NM_001172774	DPY19L3	dpy-19-like 3 (C. elegans)	
4,54	4,4	4,22	5,18	5,15	5,32	21,004	37,235	1,7728	0,001065276	NR_028370	PCNA-AS1	PCNA antisense RNA 1 (non-protein coding)	
5,78	5,97	6,02	6,6	6,71	6,93	60,8413	107,8783	1,7731	0,004113601	ENST00000364725	RN5S132	RNA, 5S ribosomal 132	
5,83	5,44	5,43	6,42	6,34	6,46	47,8027	84,89	1,7758	0,001747731	NM_018018	SLC38A4	solute carrier family 38, member 4	
6,14	6,01	6,21	7,03	6,91	6,91	69,665	123,736	1,7762	0,000266473	NM_001146276	NCEH1	neutral cholesterol ester hydrolase 1	
5,01	4,7	4,86	5,89	5,61	5,55	29,0853	51,6643	1,7763	0,006087111	ENST00000477593	PIK3CB	phosphoinositide-3-kinase, catalytic, beta polypeptide	
7,1	7	6,91	7,9	7,95	7,64	128,482	228,534	1,7787	0,002984704	NM_020354	ENTPD7	ectonucleoside triphosphate diphosphohydrolase 7	
7,99	7,72	7,76	8,67	8,6	8,72	227,2793	405,6723	1,7849	0,000437241	NM_006459	ERLIN1	ER lipid raft associated 1	
9,05	9,09	8,89	9,92	9,87	9,75	516,4757	921,868	1,7849	0,000455963	ENST00000304414	ARL6IP1	ADP-ribosylation factor-like 6 interacting protein 1	
5,07	3,88	4,4	5,47	5,37	5,26	23,142	41,3327	1,786	0,03506908	ENST00000516687	RN5S33	RNA, 5S ribosomal 33	
4,77	5,04	4,99	5,54	5,93	5,84	30,6543	54,926	1,7918	0,006476912	NR_029631	MIR218-1	microRNA 218-1	
7,12	6,87	6,79	7,82	7,78	7,77	122,2443	221,3467	1,8107	0,000376481	NM_016072	GOLT1B	golgi transport 1B	
7,03	6,73	6,97	7,96	7,69	7,65	120,7363	218,7847	1,8121	0,004412983	ENST00000222543	TFPI2	tissue factor pathway inhibitor 2	
6,78	7,06	7,03	7,49	7,87	8,05	124,674	226,246	1,8147	0,017434565	NM_031459	SESN2	sestrin 2	
5,19	4,75	5,51	6,27	5,94	5,9	36,3277	66,093	1,8194	0,018391089	NM_024518	ULBP3	UL16 binding protein 3	
4,6	4,73	4,59	5,61	5,13	5,72	24,9577	45,5223	1,824	0,019293927	ENST00000415664	TPTE	transmembrane phosphatase with tensin homology	
4,79	4,75	4,68	5,71	5,51	5,6	26,736	48,8063	1,8255	0,000421635	NM_145702	TIGD1	tigger transposable element derived 1	
4,9	4,33	4,27	5,5	5,49	5,2	23,0873	42,3183	1,833	0,011846236	ENST00000547292	LOC100506948	uncharacterized LOC100506948	
5,12	4,79	4,77	5,86	5,51	5,93	29,9083	54,8733	1,8347	0,009339764	NM_001001415	ZNF429	zinc finger protein 429	
4,52	4,3	4,58	5,51	5,46	5,03	22,1863	40,753	1,8369	0,012084566	NM_020381	PDSS2	prenyl (decaprenyl) diphosphate synthase, subunit 2	
4,72	4,47	4,33	5,58	5,28	5,32	22,8763	42,212	1,8452	0,004587825	NM_001786	CDK1	cyclin-dependent kinase 1	
5,88	5,65	5,77	6,68	6,69	6,59	54,558	100,7077	1,8459	0,00015739	NM_017423	GALNT7	UDP-N-acetyl-alpha-D-galactosamine:polypeptide N-acetylgalacto	
4,56	4,57	4,7	5,56	5,37	5,57	24,444	45,3457	1,8551	0,000618771	NM_001160183	ZNF138	zinc finger protein 138	
4,84	4,6	4,55	5,47	5,67	5,54	25,439	47,255	1,8576	0,000985696	NM_003093	SNRPC	small nuclear ribonucleoprotein polypeptide C	
5,53	5,89	5,71	6,58	6,53	6,73	52,618	98,078	1,864	0,001262663	NM_052831	SLC18B1	solute carrier family 18, subfamily B, member 1	
7,57	7,48	7,56	8,45	8,41	8,45	185,7507	346,5187	1,8655	4,86682E-06	NM_006207	PDGFRL	platelet-derived growth factor receptor-like	
5,02	5,17	5,13	5,99	6,05	5,99	34,4887	64,4577	1,869	2,73799E-05	NM_181785	SLC46A3	solute carrier family 46, member 3	
5,86	5,66	5,97	6,67	6,74	6,81	57,109	106,9753	1,8732	0,000421998	ENST00000374692	TMEM38B	transmembrane protein 38B	
7,32	7,03	7,16	8,04	8,06	8,14	144,4963	270,7187	1,8735	0,000247398	NM_022166	XYLT1	xylosyltransferase I	
4,72	4,83	4,32	5,8	5,56	5,22	24,9237	46,721	1,8746	0,021015553	AK126318	FLJ44342	uncharacterized LOC645460	
6,25	5,48	6,15	6,64	6,87	7,18	63,9177	120,5707	1,8863	0,025987843	NR_024241	FAM86DP	family with sequence similarity 86, member D, pseudogene	
6,37	6,83	6,57	7,5	7,55	7,51	97,1643	183,5667	1,8892	0,000728217	NM_003842	TNFRSF10B	tumor necrosis factor receptor superfamily, member 10b	
6,51	6,2	6,05	7,25	7,08	7,26	76,971	146,9313	1,9089	0,001745951	NM_152729	NT5DC1	5'-nucleotidase domain containing 1	
4,55	4,81	4,36	5,31	5,56	5,67	24,0037	45,9207	1,9131	0,005231657	NM_024808	BORA	bora, aurora kinase A activator	
4,83	4,75	4,92	5,66	5,93	5,74	28,542	54,9927	1,9267	0,001241258	NM_024430	PSTPIP2	proline-serine-threonine phosphatase interacting protein 2	
5	5,18	5,27	6,1	6,05	6,18	35,6127	69,1187	1,9408	0,000226315	NM_020663	RHOJ	ras homolog family member J	
5,81	5,88	5,87	6,77	6,94	6,72	57,8267	112,4477	1,9446	0,00051882	NM_016027	LACTB2	lactamase, beta 2	
6,02	6,14	6,11	7,05	6,99	7,13	68,162	133,2333	1,9547	9,37449E-05	NM_201649	SLC6A9	solute carrier family 6 (neurotransmitter transporter, glycine), mem	
5,39	5,29	5,3	6,32	6,31	6,26	40,1513	78,6243	1,9582	8,90484E-06	ENST00000481661	GPR135	G protein-coupled receptor 135	

Pre-miR-Ctrl (Log2)			Pre-miR-132 (Log2)			Average		Fold Change (miR-132 vs Ctrl)	p-value	Gene Assignment			Predicted miR-132 binding sites
Sample 1	Sample 2	Sample 3	Sample 1	Sample 2	Sample 3	Pre-miR-Ctrl	Pre-miR-132			Public id	Symbol	Name	
4,36	5,01	4,75	5,57	5,92	5,63	26,5557	52,525	1,9779	0,007922286	NM_001195056	DDIT3	DNA-damage-inducible transcript 3	
6,07	6,3	6,13	7,16	7,22	7,13	72,0033	144,0563	2,0007	8,04851E-05	ENST00000394053	MTHFD2	methylenetetrahydrofolate dehydrogenase (NADP+ dependent) 2	
5,52	5,42	5,67	6,56	6,58	6,49	46,5383	93,3023	2,0048	9,12251E-05	NM_000153	GALC	galactosylceramidase	
5,5	4,22	4,62	5,96	5,87	5,89	29,4937	60,0123	2,0348	0,020012959	NR_033801	SLC9A7P1	solute carrier family 9, subfamily A (NHE7, cation proton antiporte	
4,7	4,7	4,57	5,76	5,34	5,91	25,2453	51,6083	2,0443	0,010822564	NM_030919	FAM83D	family with sequence similarity 83, member D	
5,98	5,85	5,72	6,86	6,95	6,85	57,8363	118,3873	2,0469	0,000110826	NM_018394	ABHD10	abhydrolase domain containing 10	
5,88	5,69	5,97	6,86	6,88	6,93	57,7333	118,628	2,0548	7,74472E-05	NM_016399	TRIAP1	TP53 regulated inhibitor of apoptosis 1	
5,46	5,38	5,3	6,42	6,3	6,57	41,6857	86,4767	2,0745	0,000786324	NM_203394	E2F7	E2F transcription factor 7	
5,44	4,99	5,04	6,25	6,09	6,33	36,03	74,8927	2,0786	0,001680358	ENST00000377103	THBD	thrombomodulin	
4,47	4,5	4,02	5,36	5,66	5,15	20,3373	42,3797	2,0838	0,010496456	NM_001204818	ZNF587B	zinc finger protein 587B	
6,4	5,96	6,21	7,28	7,33	7,2	73,5757	154,4493	2,0992	0,000435847	ENST00000264107	ITGA6	integrin, alpha 6	
6,02	5,67	5,85	6,87	7,02	6,89	57,829	121,7867	2,106	0,000359781	NM_005729	PPIF	peptidylprolyl isomerase F	
4,72	4,98	4,59	6,02	5,61	5,91	27,3327	57,954	2,1203	0,004302914	ENST00000359193	HIST1H2AH	histone cluster 1, H2ah	
4,48	3,97	4,48	5,03	5,65	5,53	20,101	43,0303	2,1407	0,016300496	NM_030763	HMGN5	high mobility group nucleosome binding domain 5	
8,93	9,26	8,93	10,16	10,2	10,18	529,537	1160,1473	2,1909	0,000123558	NM_058179	PSAT1	phosphoserine aminotransferase 1	
5,91	5,99	5,69	7,07	6,91	7,02	58,4373	128,1367	2,1927	0,000217426	NM_138455	CTHRC1	collagen triple helix repeat containing 1	
5,84	6,58	6,19	7,37	7,43	7,34	75,3203	166,628	2,2123	0,001388356	NM_014278	HSPA4L	heat shock 70kDa protein 4-like	
5,57	5,39	5,75	6,88	6,62	6,66	47,7517	105,7563	2,2147	0,001139344	NM_002203	ITGA2	integrin, alpha 2 (CD49B, alpha 2 subunit of VLA-2 receptor)	
5,57	6,04	5,98	6,95	7,1	7,19	58,8077	135,615	2,3061	0,000890113	ENST00000446533	CHAC1	ChaC, cation transport regulator homolog 1 (E. coli)	
8,31	8,11	7,84	9,25	9,27	9,4	274,2577	633,9453	2,3115	0,000402571	ENST00000394485	MT1X	metallothionein 1X	
5,28	5,34	5,49	6,41	6,78	6,56	41,4333	96,4283	2,3273	0,001818562	ENST00000325617	CLGN	calmegin	
6,25	6,78	6,72	7,64	7,93	7,93	97,1417	229,072	2,3581	0,001924189	NM_019058	DDIT4	DNA-damage-inducible transcript 4	
5,48	5,62	5,64	7,02	6,77	6,7	47,893	114,2973	2,3865	0,001181645	NM_145172	WDR63	WD repeat domain 63	
4,77	4,73	4,08	5,64	6,09	5,73	23,578	57,021	2,4184	0,006927928	NM_003383	VLDLR	very low density lipoprotein receptor	
4,77	5,05	4,8	6,36	5,96	6,26	29,4233	73,676	2,504	0,002055539	NM_001902	CTH	cystathionase (cystathionine gamma-lyase)	
6,4	6,32	6,14	7,67	7,66	7,67	78,288	203,1883	2,5954	7,09618E-06	NM_176870	MT1M	metallothionein 1M	
9,28	8,91	9,14	10,71	10,43	10,52	555,6263	1507,6667	2,7135	0,000594246	NM_002421	MMP1	matrix metallopeptidase 1 (interstitial collagenase)	
5,62	5,2	5,32	6,87	6,95	6,98	41,9617	122,2827	2,9142	6,51343E-05	NM_006727	CDH10	cadherin 10, type 2 (T2-cadherin)	
4,82	4,27	5,09	6,66	6,24	5,96	27,1997	79,653	2,9285	0,012605385	NR_003224	SNORD114-31	small nucleolar RNA, C/D box 114-31	
